# Monocyte Chemoattractant Protein-Induced Protein 1 (MCPIP1) Enhances Angiogenic and Cardiomyogenic Potential of Murine Bone Marrow-Derived Mesenchymal Stem Cells

**DOI:** 10.1371/journal.pone.0133746

**Published:** 2015-07-27

**Authors:** Anna Labedz-Maslowska, Barbara Lipert, Dominika Berdecka, Sylwia Kedracka-Krok, Urszula Jankowska, Elzbieta Kamycka, Malgorzata Sekula, Zbigniew Madeja, Buddhadeb Dawn, Jolanta Jura, Ewa K. Zuba-Surma

**Affiliations:** 1 Department of Cell Biology, Faculty of Biochemistry, Biophysics and Biotechnology, Jagiellonian University, Krakow, Poland; 2 Department of General Biochemistry, Faculty of Biochemistry, Biophysics and Biotechnology, Jagiellonian University, Krakow, Poland; 3 Department of Physical Biochemistry, Faculty of Biochemistry, Biophysics and Biotechnology, Jagiellonian University, Krakow, Poland; 4 Malopolska Centre of Biotechnology, Jagiellonian University, Krakow, Poland; 5 Division of Cardiovascular Diseases, Cardiovascular Research Institute, University of Kansas Medical Center, Kansas City, Kansas, United States of America; Northwestern University, UNITED STATES

## Abstract

The current evidence suggests that beneficial effects of mesenchymal stem cells (MSCs) toward myocardial repair are largely due to paracrine actions of several factors. Although Monocyte chemoattractant protein-induced protein 1 (MCPIP1) is involved in the regulation of inflammatory response, apoptosis and angiogenesis, whether MCPIP1 plays any role in stem cell-induced cardiac repair has never been examined. By employing retroviral (RV)-transduced overexpression of MCPIP1, we investigated the impact of MCPIP1 on viability, apoptosis, proliferation, metabolic activity, proteome, secretome and differentiation capacity of murine bone marrow (BM) - derived MSCs. MCPIP1 overexpression enhanced angiogenic and cardiac differentiation of MSCs compared with controls as indicated by elevated expression of genes accompanying angiogenesis and cardiomyogenesis *in vitro*. The proangiogenic activity of MCPIP1-overexpressing MSCs (MCPIP1-MSCs) was also confirmed by increased capillary-like structure formation under several culture conditions. This increase in differentiation capacity was associated with decreased proliferation of MCPIP1-MSCs when compared with controls. MCPIP1-MSCs also expressed increased levels of proteins involved in angiogenesis, autophagy, and induction of differentiation, but not adverse inflammatory agents. We conclude that MCPIP1 enhances endothelial and cardiac differentiation of MSCs. Thus, modulating MCPIP1 expression may be a novel approach useful for enhancing the immune-regulatory, anti-apoptotic, anti-inflammatory and regenerative capacity of BM-derived MSCs for myocardial repair and regeneration of ischemic tissues.

## Introduction

Bone marrow (BM)-derived mesenchymal stem cells (MSCs) have been used extensively for tissue regeneration due to their broad differentiation potential, low immunogenicity and innate anti-inflammatory properties [1–3]. It has also been reported that a fraction of MSCs are able to differentiate into cardiomyocytes and endothelial cells following stimulation *in vitro* or *in vivo* [4–9]. Several recent studies indicate that therapy with BM-derived MSCs improves left ventricular (LV) function and myocardial perfusion after myocardial infarction (MI) [1, 10–12]. However, the benefits of MSC therapy for cardiac repair *in vivo* has been variable [1, 10].

Therefore, several approaches have been employed to enhance the capacity of MSCs for ischemic tissue repair. These include overexpression of multiple exogenous factors, including anti-apoptotic and pro-surviving proteins (e.g. Hsp20, Hsp27, survivin) [13–15] as well as growth factors with pleiotropic effects, including proangiogenic activities (e.g. vascular endothelial growth factor (VEGF), hepatocyte growth factor (HGF), angiopoietin-1, glycogen synthase kinase-3β (GSK-3β), sonic hedgehog (Shh)) [16–20]. Although such strategies have been attempted for many years, there is still no optimized set of factors or individual molecule that may definitively augment the reparative properties of MSCs and enhance cardiac repair.

Monocyte Chemoattractant Protein-1–Induced Protein 1 (MCPIP1; Zc3h12a) has been identified in human macrophages following stimulation with interleukin 1β (IL-1β) [21]. Although the highest level of MCPIP1 has been found in leukocytes, it may also be expressed in other cell types [21]. MCPIP1 has been shown to be induced by several proinflammatory agents and cytokines, and may act as a macrophage activator and negative regulator of oxidative stress and inflammatory gene expression [22]. Moreover, overexpression of MCPIP1 in these cells significantly decreased promoter activity of tumor necrosis factor α (TNF-α) and inducible nitric-oxide synthase (iNOS) in a dose-dependent manner, indicating anti-inflammatory properties [22]. Interestingly, it has recently been shown that MCPIP-1 exhibited RNAse activity due to the presence of a PilT N-terminus (PIN) domain and may play a regulatory (including anti-inflammatory) role via mRNA and pre-miRNA decay in different cell types, including non-hematopoietic cells [21, 23, 24]. Importantly, Niu and colleagues have reported that MCPIP1 may be involved in enhancing angiogenic activity and secretion of several proangiogenic factors in human umbilical vein endothelial cells (HUVECs) [25]. Moreover, the specific knockdown of MCPIP1 in these cells resulted in suppression of VEGF and hypoxia-inducible factor-1α (HIF-1α), which are important agents involved in proangiogenic and antioxidative cell responses [25]. Notably, studies employing transgenic mice with myocardial MCPIP1 expression (under an α-MHC promoter) have reported extensive attenuation of inflammatory response-related cardiac dysfunction [26]. Data from several laboratories strongly indicate that MCPIP1 may reside in both cytoplasmic as well as nuclear compartments with distinct roles in different cell types [21, 23, 27].

Although the impact of MCPIP1 on angiogenic potential, survival and inflammatory response was studied in several mature cell types, the role of MCPIP1 in stem cells, such as MSCs has never been investigated. Thus, in this study we evaluated the impact of MCPIP1 on several functions of MSCs, including cell viability and apoptosis, proliferation, metabolic activity, transcriptomic, proteome and secretome profiles, as well as angiogenic and cardiomyogenic differentiation capacity.

## Materials and Methods

### Animals

Four-to-six-week-old C57Bl/6 mice were used for experiments. All procedures were performed in accordance with the approval of the Ethical Committee on Animal Testing at the Jagiellonian University (JU) in Krakow (approval number: 31/2012). C57Bl/6 mice were supplied for experiments by Charles River (Wilmington MA, USA) and were subsequently held for 7 day quarantine, prior to their experimental use, in Animal Facility of Department of Biophysics at the Faculty of Biochemistry, Biophysics and Biotechnology JU in Krakow. Mice were handled accordingly to standard animal procedures complied in controlled animal facilities. Up to 5 mice of the same gander were kept per one cage and were constantly controlled for health conditions, supplied with water and dry fodder containing balanced nutrition and were handled accordingly to the standard regulations. Animals were sacrificed by intraperitoneal injection of lethal dose of pentobarbital (Morbital; Biowet, Pulawy, Poland; 100mg/kg b.w.) representing acceptable method for mice euthanasia and accordingly to the ethical approval listed above. Tibias and femurs were harvested immediately following the animal euthanasia.

### Isolation and culture of MSCs

Bone marrow cells were harvested by flushing cavities of tibia and femur bones with DMEM/F12 medium (Sigma-Aldrich). Cells were centrifuged, re-suspended in complete medium (DMEM/F12 with 10% FBS, Sigma-Aldrich; and penicillin/ streptomycin, Gibco, Life Technologies) and seeded into a Primaria culture flask (BD Falcon) at a density of 25x10^6^ nucleated cells/ 75cm^2^. Flushed bones were additionally fragmented and enzymatically digested with collagenases type I and II (1 mg/ml; Sigma Aldrich) for 1.5h at 37°C. Released cells were washed and added equally to flasks containing flushed BM cells. Cells were cultured in standard conditions for 72h and non-adherent cells were removed. Cells were passaged with 0.25% trypsin/ EDTA (Gibco, Life Technologies) when confluence of cells reached close to 90%.

### Retrovirus generation

Retroviral vectors containing the following plasmids were prepared in our laboratory and used in the study: pMX-MCPIP1 coding for MCPIP1, pMX-GFP (control plasmid containing insert with enhanced green fluorescence protein (GFP)), and pMX-Puro (empty plasmid, devoid of any insert). Retrovirus packaging was performed in a modified HEK293 (Phoenix Amphotropic) as described earlier [28].

### Retroviral transduction

To obtain MCPIP1 overexpressing MSCs, retrovirus-mediated gene transfer based on pMX-Puro system was performed [29]. MSCs on passage 3 to 4 were transduced with vectors: pMX-MCPIP1, pMX-GFP or pMX-Puro. Each vector was added to 0.5x10^6^ MSCs in the presence of 4 μg/ml polybrene (Merck Millipore) in complete growth medium (MOI = 7). The medium was changed after 24h. In order to increase the population of MCPIP1-overexpressing cells, transduction was repeated after 48h.

### Viability, proliferation and metabolic activity assessment

The viability and metabolic status of MSCs were measured by MTT assay and ATP concentration, respectively. Tests were performed at 48 and 72h after second transduction.

### MTT assay

Cells were seeded on transparent 96-well plates (BD Falcon) to reach 90% of confluence. Then MTT (500 ng/ml; Sigma-Aldrich) was added for 4h. The medium was removed and acidic isopropanol (40 mM HCl; POCh) was added for 30 min. Absorbance was measured with an Infinite 200 microplate reader (Tecan Group Ltd.) at a wavelength of 570 nm with background subtraction at 650 nm.

### ATP concentration measurement

MSCs were seeded on 96-well white plates to obtain 90% of confluence in each time point and the ATP Lite Luminescence assay kit was performed according to manufacturer’s instructions (Perkin Elmer). Luminescence was measured using the SpectraFluor Plus (Tecan Group Ltd) microplate reader.

### Proliferation ratio assessment

The relative proliferation ratio of MSCs was evaluated at 72h after the second transduction. MSCs were counted with the Countess II Automated Cell Counter (Life technologies).

### Western blotting

Expression of MCPIP1 at the protein level was detected by western blotting [30]. Briefly, MCPIP1 was assessed using primary rabbit anti-MCPIP1 antibody [21]. As a loading control, primary mouse anti-actin antibody (Sigma-Aldrich) was used. Detailed procedure is included in S1 Appendix (Extended Materials and Methods).

### Antigenic phenotyping by flow cytometry

The phenotype of MSCs was evaluated at 72h after the second transduction. MSCs were immunolabelled with the following monoclonal antibodies: anti-CD45 (APC-Cy7 or FITC, clone: 30-F11, BD Bioscence), anti-Sca-1 (PE, clone: E13-161.7, BD Bioscence), anti-CD105 (PE/Cy7, clone: MJ7/18, BioLegend), and anti-CD90.2 (APC, clone: 30-H12, BioLegend). Staining was performed according to manufacturer’s protocols for 30 min at 4°C. Cells were analyzed using LSR II flow cytometer and FACS Diva software (Becton Dickinson).

### Necrosis and apoptosis detection

Assessment of necrosis and apoptosis was done with Annexin V Apoptosis Detection Kit (BD Biosciences) and by Vybrant FAM Caspase-3 and 7- Assay Kit (Life Technologies) with flow cytometry. Staining was performed according to manufacturer’s protocols and analyzed using using LSR II flow cytometer and FACS Diva software (Becton Dickinson).

### Global proteomic analysis—sample processing

MSCs (1.7 x 10^6^) from all groups were lysed in 4% SDS and 0.1 M DTT in Tris-HCl (BioShop, pH 7.6) and sonicated for 10 min. The samples were further incubated at 95°C for 5 min and centrifuged at 30,000*g* for 30 min at 20°C. Then, the proteins were digested using the filter aided sample preparation (FASP) technique [31] (detailed procedures including liquid chromatography and tandem mass spectrometry as well as analysis of proteomic data are included in S1 Appendix (Extended Materials and Methods).

### Capillary-like tube formation assay

Twenty four-well plates were coated with Matrigel Matrix Grow Factor Reduced (BD Pharmingen) (100μl/well) and incubated at 37°C for 30 min. MCPIP1-expressing MSCs and control MSCs were seeded at a density of 1x10^5^ cells/well in the EGM-2MV medium (Clonetics, Lonza). HUVECs were used as a positive control, while freshly isolated total nucleated cells (TNCs) from murine BM were utilized as a negative control. Cells were incubated for 12 or 18h at 37°C. Tube formation was investigated every 2h and images were collected with an Olympus IMT-2 microscope equipped with a CCD camera. The results were computed as absolute number of capillary-like structures and branches per field formed by cultured cell groups at different time points along the assay.

### Cardiomyogenic and angiogenic differentiation

#### Cardiomyogenic differentiation

MSCs were cultured on a dish coated with 50 μg/ml collagen type I (Sigma-Aldrich) in DMEM/F12 with 2% FBS (Sigma-Aldrich) and 10ng/ml bFGF, 10ng/ml VEGF and 10 ng/ml TGFβ1 [32] (all growth factors from R&D Systems). Cells were examined for cardiac differentiation after 5 and 10 days of culture.

#### Endothelial differentiation

MSCs were cultured on a dish coated with both fibronectin (50 μg/ml, Corning) and gelatin (0.1%, Sigma-Aldrich) in EGM-2MV endothelial medium (Lonza). Cells were examined for angiogenic differentiation after 5 and 10 days of culture.

### Gene expression analysis by quantitative real-time RT-PCR

Total RNA was isolated with GeneMATRIX Universal RNA Purification Kit (EURx) and 200ng of total RNA was used for reverse transcription with TaqMan Reverse Transcription Reagents (Life Technologies) performed according to the manufacturer’s protocol. Expression of genes related to pluripotency state (Oct-3/4A, Sox2, Klf4, c-Myc), cardiac (Gata-4, Nkx2.5, Myl2, Myh6) and endothelial (Gata-2, Tie-2, VE-cadherin, vonWillebrand factor (vWF)) differentiation were examined by real-time PCR using an ABI PRISM 7000 system (Applied Biosystems). β2-microglobulin was used as a control housekeeping gene.

Real-time PCR was performed using Sybr Green qPCR Master Mix (EURx), cDNA template (10ng), forward and reverse primer (1μM; Genomed). The sequences of primers are included in S1 Appendix (Extended Materials and Methods). Relative quantification of gene expression was calculated using the comparative C_t_ method. The relative quantitative value of the target normalized to an endogenous control (β2-microglobulin gene) and relative to a calibrator was expressed as 2^-ΔΔCt^ (i.e.-fold difference).

### Immunocytochemistry

Immunocytochemistry staining was performed to evaluate: i) Gata-4 and troponin T-C; and ii) Gata-2 and VE-cadherin expression in MSC-derived cardiac and endothelial cells, respectively. Cells were fixed with 4% paraformaldehyde, permeabilized with 0.1% Triton X-100, washed, and stained with primary anti-Gata-4 and anti-troponin T-C antibodies or with anti-Gata-2 and anti-VE-cadherin antibodies followed by secondary antibodies and DAPI. Cells were analyzed with a Leica DM IRE2 (Ver. 4000) fluorescent microscope. A detailed description of the staining procedure and used antibodies are included in S1 Appendix (Extended Materials and Methods).

### Secretome analysis

#### Western blotting (semi-quantitative)

MCPIP1-expressing MSCs and control cells at 72h following the second transduction were cultured in EGM-2MV medium for 10 days and subsequently were washed twice with DMEM/F12 and further cultured in DMEM/F12 with 0.5%BSA (Sigma-Aldrich) for 24h. Cell culture supernatants were collected and frozen at -80°C until analysis. Analysis of expression of 53 angiogenesis-related proteins was carried out using the Proteome Profiler Mouse Angiogenesis Array Kit (R&D Systems) according to the manufacturer’s protocol. The average signals (pixel density) computed from duplicate spots representing each angiogenesis-related protein were determined by Quantity One software (BioRad). An average background signal was subtracted from each spot during the analysis.

#### Multiplex analysis (quantitative)

In addition, cell culture supernatants were evaluated for the presence of selected angiogenesis- related factors with a Luminex-based platform. Quantitative analysis of concentrations of endoglin, endothelin, VEGF-α, HGF, SDF-1, MCP-1 and IL-1β was performed using the Milliplex MAP Kit (Mouse Angiogenesis/Growth Factor Magnetic Bead Panel; EMD Millipore) according to the manufacturer’s protocol. Protein concentrations were read on a Luminex platform (Austin). Median Fluorescent Intensity (MFI) for each protein was analyzed using 5-parameter logistic or spline curve-fitting method for calculating concentrations in tested samples by in Luminex IS 2.3 Software (Austin).

### Statistical analysis

All experiments were carried out in triplicates (except global proteome analysis which was repeated twice). In every repetition of the experiment was used primary culture of MSCs at passage 3–4. Results are presented as mean values ± standard deviations (SD). Paired Student’s *t* tests were used to compare MCPIP1-overexpressing MSCs and Puro-treated cells. Untreated Control cells were used in all experiments as a negative control. A P value <0.05 was considered as statistically significant. All statistical analyses were performed using the Origin (ver. 9.1) statistical software (Microcal Software).

## Results

### MCPIP1 is efficiently overexpressed in primary MSCs following retroviral transduction

To evaluate the impact of MCPIP1 on functions of BM-derived MSCs, cells were transduced with retroviral vectors carrying the sequence coding MCPIP1 (MCPIP1-MSCs). Empty vector-treated (Puro) or untreated (Control) MSCs were used as controls. In order to confirm expression of MCPIP1 at the mRNA level, total RNA was isolated from MSCs at 48 and 72 hours after transduction. The relative level of expression of mRNA for MCPIP1 was computed in comparison with Puro-treated cells (set as 1) (S1C Fig). The expression of transcript in MCPIP1-MSCs was higher in comparison with control cells at each time point. However, the highest concentration of mRNA for MCPIP1 was observed at 72h post transduction in MCPIP1-MSCs (S1C Fig), while the transcript was hardly detectable in untreated Control cells. The expression was also confirmed on protein level (S1D Fig).

To investigate the potential impact of MCPIP1 on MSC viability and metabolic activity, several functional tests were done at the indicated time points post transduction. Thus, MTT assay was used to measure cytotoxic effects of MCPIP1 at 48 and 72h following viral transduction. Relative viability was computed when compared with Puro-treated cells (shown as 1). We did not observe any notable impairment of cell viability after transduction with retroviral vectors at each time point (S1E Fig). The effect of MCPIP1 on MSC metabolic activity was evaluated by the ATP measuring assay. Relative metabolic activity was also computed in comparison to Puro-treated cells (set as 1). We did not find a significant impact of MCPIP1 protein on metabolic activity of MSCs at any time-point (S1F Fig).

The transduction efficiency was evaluated following MSC treatment with a retroviral vector carrying GFP for 72h by applying both fluorescent microscopy and flow cytometry (S1A and S1B Fig). A total of 60.0 ± 16.5% of transduced adherent fraction of cultured BM cells expressed GFP, corresponding to transduction efficiency within the entire fraction of cells (S1B Fig).

Thus, because of the highest expression of MCPIP1 observed at 72h post transduction that was not accompanied by significant impairment in viability or metabolic activity of MCPIP1- overexpressing MSCs, the cells at this time-point after transduction were subsequently used for further experiments examining the impact of MCPIP1 on several MSC functions including SC-related gene expression as well as angiogenic and cardiomyogenic differentiation potential *in vitro*.

### MCPIP1 does not affect antigenic phenotype and viability but decreases MSC proliferation

**Fig 1:**
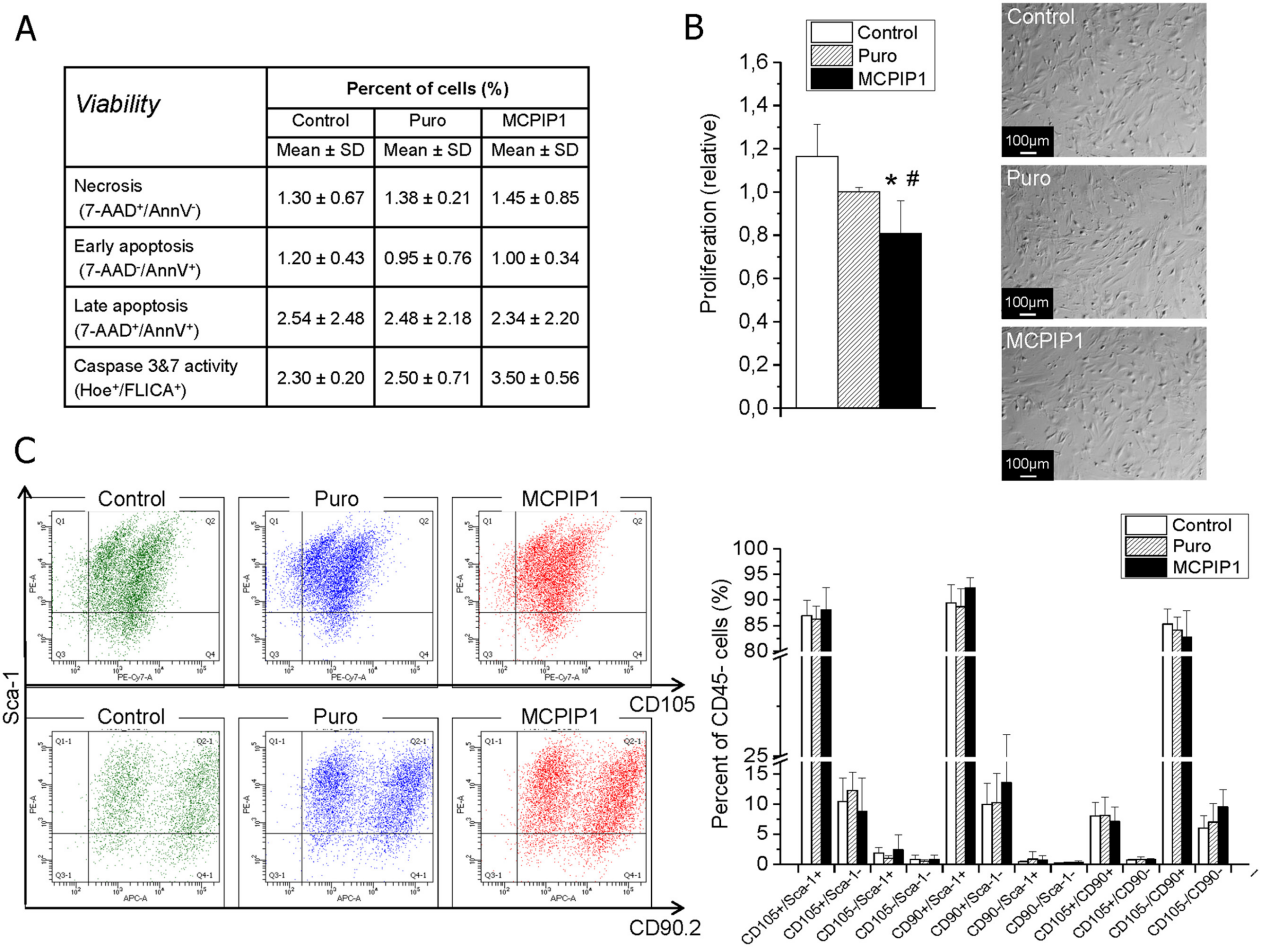
Impact of MCPIP1 expression on selected functions of MSCs at 72h post transduction. **(A)** Viability and apoptosis induction by flow cytometry assays. Table represents percent content of cells undergoing necrosis, early apoptosis, late apoptosis and exhibiting caspase 3 and 7 activation among MCPIP1- overexpressing MSCs, empty vectors (Puro)- treated and untreated (Control) MSCs. **(B)** Proliferation by Countess II Automated Cell Counter (Life Technologies) (left). The graph shows the relative level of proliferation of MCPIP1-overexpressing MSCs (black bar) when compared with Puro-treated cells (hatched bar; recalculated as 1) and untreated Control MSCs (white bar). Morphology of MCPIP1- overexpressing MSCs, empty vectors (Puro)- treated and untreated (Control) MSCs (right). Scale bars: 100 μm. **(C)** Antigenic profile of MSCs by flow cytometry. Expression of CD90, CD105 and Sca-1 antigens on MCPIP1-overexpressing MSCs and control cells (Control and Puro) is shown on representative dot-plots. Analyzes were performed on CD45- subsets indicating MSCs using the LSR II flow cytometer (Becton Dickinson). Right graph shows quantitative data representing percent content of each subpopulation of antigenically-defined MSCs among three experimental groups. All results are presented as mean ± SD. Statistically significant differences (P<0.05) are shown when compared with Puro (*) and Control (#). Analysis based on three independent experiments. Control—untreated MSCs; Puro—empty vector-treated MSCs; MCPIP1- MSCs overexpressing MCPIP1.

To evaluate the impact of MCPIP1 on MSC viability and apoptosis activation, several assays were done at 72h post transduction. Apoptosis and necrosis were examined by phosphatydyloserine detection and 7-aminoactinomycin D staining as well as by evaluation of caspase-3 and -7 activation. No major impact of MCPIP1 on MSC viability was noted. A small fraction of cells (less than 5%) undergoing cell death was detected within in all groups of MSCs (Fig 1A).

We also evaluated the impact of MCPIP1 on proliferation of MSCs at 72h after transduction. Interestingly, we found that MCPIP1 overexpression was associated with a significant decrease in MSC proliferation rate when compared with both control groups (Fig 1B). However, the proliferative activity of MSCs overexpressing MCPIP1 was still sustained at a relatively high level (80.19% ± 7.34 when compared with Puro-treated cells). Importantly, we did not observe any major differences in morphology between MCPIP1-overexpressing MSCs and control cells (Fig 1B).

To investigate the potential influence of MCPIP1 overexpression on expression of classical MSC markers and the content of major antigenically- defined MSC subpopulations, we evaluated the multiantigenic profile of transduced cells by immunostaining against CD45, CD90.2, CD105 and Sca-1 antigens followed by flow cytometric analysis (Fig 1C). We found that similar to control cells, MCPIP1-overexpressing MSCs consist of three main subpopulations including CD90^+^/CD105^-^, CD105^+^/Sca-1^+^ and CD90^+^/Sca-1^+^ that represent 82.7±5.2%; 88.1±4.4% and 92.4±2.0% of CD45-negative MSC population, respectively (Fig 1C). Furthermore, no major effect on expression of MSC-related surface antigens was observed in cells with MCPIP1 overexpression. Thus, the content of main subsets of MSCs was comparable between MCPIP1-overexpressing cells and both control groups (Fig 1C).

### MCPIP1 decreases expression of pluripotency-associated genes and enhances differentiation capacity of MSCs

**Fig 2:**
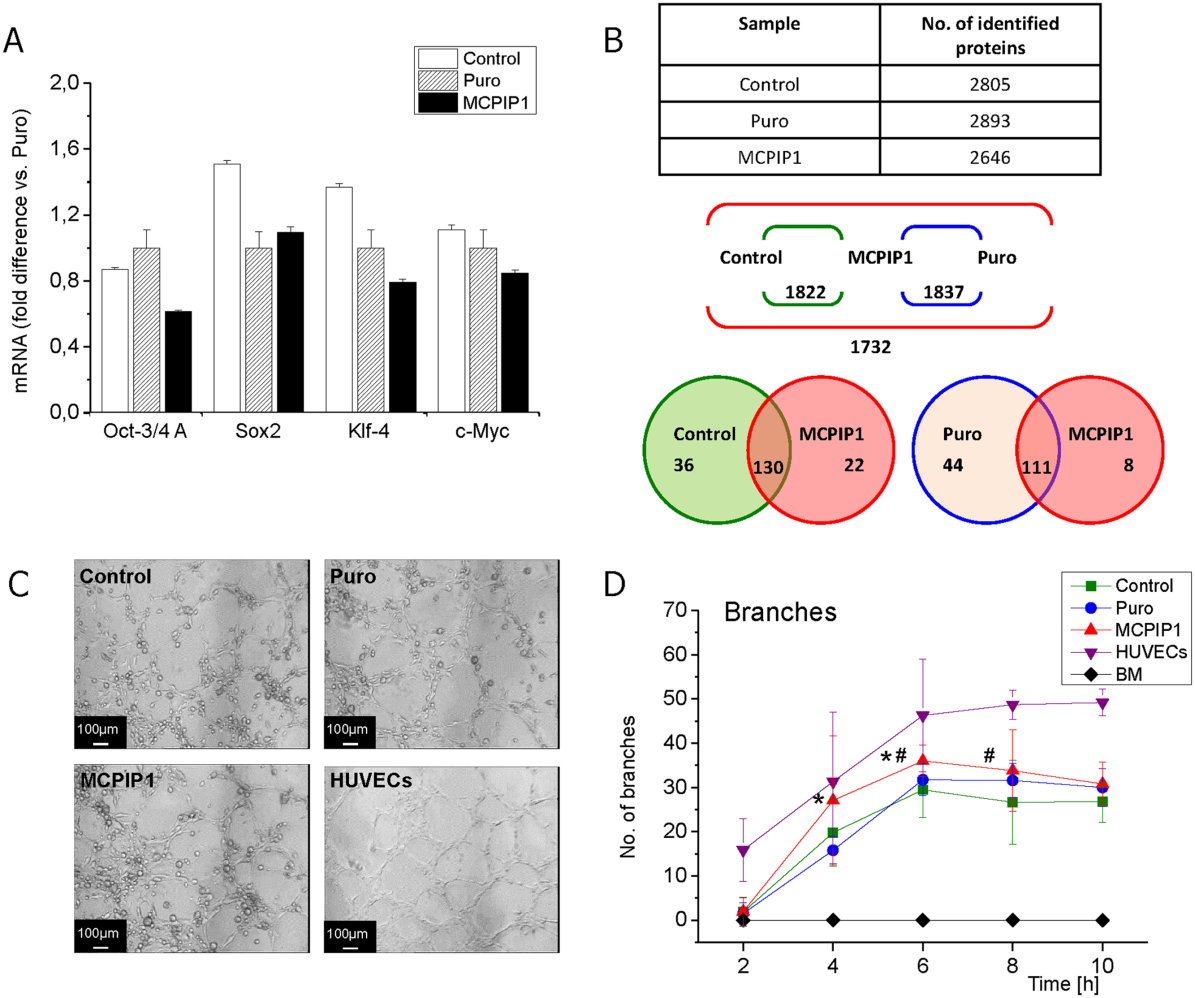
Stem cell-related genes and differentiation capacity of MSCs at 72h post transduction. **(A)** Quantitative analysis of mRNA expression for pluripotency related genes by real-time RT-PCR. The graph shows expression of Oct-4, Klf-4, Sox2 and c-Myc in MCPIP1-overexpressing MSCs and control cells (Puro, Control). Fold change in mRNA concentration was computed using the ddCt method when compared with Puro-treated cells (shown as 1). **(B)** Global proteomic analysis of MSCs at 72h post transduction by mass spectroscopy. Upper panel- Average number of proteins identified in three MSC groups: MCPIP1-overexpressing MSCs, empty vector (Puro)-treated MSCs and untreated (Control) MSCs. Middle panel- Scheme representing total number of common proteins identified in pairs: MCPIP1-overexpressing MSCs vs. Control and MCPIP1-overexpressing MSCs vs. empty vector (Puro)- treated MSCs; and number of common proteins that occur in all analyzed samples. Lower panel- Scheme showing number of proteins identified exclusively in each experimental group as well as common proteins for all compared groups with dNAFs fold change in expression higher than 2.0. **(C)** The angiogenic potential of MSCs determined by capillary-like tube formation assay. Photos show representative images of capillary- like structures formed on matrigel by MCPIP1- overexpressing MSCs as well as Puro- treated and untreated Control MSCs. Scale bars: 100 μm. **(D)** Quantitative analysis of angiogenic potential of MSCs overexpressing MCPIP1 when compared with control cells. Graphs represent number of branches at 2, 4, 6, 8 and 10h of capillary formation assay on matrigel. Six randomly selected images of high-power fields for every experimental timepoint were included in quantitative analysis. HUVEC cells were used as positive control, while freshly isolated BM cells represent the negative control. Numbers of branches formed by each cell fraction were computed based on microscopic images as shown in S2B Fig, and the detailed results (Mean number ± SD) are included in S1 Table. All results are presented as mean ± SD. Statistically significant differences (P<0.05) are shown when compared with Puro (*) and Control (#). Analysis based on three independent experiments. Control—untreated MSCs; Puro—empty vector-treated MSCs; MCPIP1- MSCs overexpressing MCPIP1.

The expression of pluripotency markers such as Oct-3/4A, Klf-4, c-Myc and Sox2 was evaluated at the mRNA level by real-time RT-PCR (Fig 2A) to assess whether MCPIP1 may be involved in regulation of stem cell-related genes. The results showed that MCPIP1 overexpression was associated with a decreased level of Oct-4 and Klf-4 (transcription factors maintaining *stemness*) in comparison with Puro-treated and untreated Control MSCs (Fig 2A).

Importantly, the Matrigel assay showed that the decrease in SC- related gene expression was accompanied by enhanced angiogenic differentiation capacity of MSCs expressing MCPIP1 when compared with control groups of MSCs (Fig 2C and 2D). In quantitative analysis (S1B Fig), we observed faster and greater formation of capillary-like structures by MSCs expressing MCPIP1 when compared with control cells (Fig 2C and 2D, S1 Table). The results suggest a role for MCPIP1 in enforcing the differentiation capacity of MSCs.

Interestingly, the proteomic data may also suggest that MCPIP1 expression favors decreased proliferative status of MSCs along with promoting their differentiation ability. Mass spectroscopy was used to evaluate the global proteome of all examined groups of MSCs. In each analyzed sample were detected more than 2600 proteins (Fig 2B). We found several proteins uniquely expressed or with elevated concentration in MCPIP1- expressing MSCs when compared with Puro-treated cells, including proteins involved in cell proliferation, differentiation, Wnt/β-catenin signaling as well as regulating processes of apoptosis and autophagy (Table 1). Interestingly, several proteins negatively regulating cell proliferation were present in this group including testin and protein phosphatase 1G implicated in negative regulation of cell proliferation and cell cycle arrest, respectively (Table 1). Moreover, we identified several proteins not present in MCPIP1-overexpressing MSCs when compared with Puro cells, including proteins negatively regulating Notch signaling as well as involved in apoptosis and intracellular reorganization (Table 2). Thus, the proteomic profile of MCPIP1-overexpressing MSCs corresponds to their lower proliferative activity and enhanced differentiation capacity.

**Table 1:** Proteins unique or greatly expressed in MCPIP1- expressing MSCs when compared with Puro-treated MSCs.

Proteins unique for MCPIP1-overexpressing MSCs			
*Accession number*:	*Protein name*	*Fold change*	*Selected function [Based on UniProtKB]*
Q5D1E8	Ribonuclease ZC3H12A	ND	Pro-inflammatory cytokines mRNA decay; angiogenesis
Q9CQB5	CDGSH iron-sulfur domain-containing protein 2	ND	Autophagy regulation
P62257	Ubiquitin-conjugating enzyme E2 H	ND	Protein ubiquitination
Q6PFQ7	Ras GTPase-activating protein 4	ND	Ras protein signal transduction regulation
P70444	BH3-interacting domain death agonist	ND	Caspases and apoptosis induction
Q8K3C3	Protein LZIC	ND	Wnt/ β-catenin signaling
Q3UL36	Arginine and glutamate-rich protein 1	ND	ER-mediated transcription; required for cell growth
**Quantitatively more in MCPIP1-overexpressing MSCs when compared with Puro**			
O35509	Ras-related protein Rab-11B	6.71	Key regulators of intracellular membrane trafficking
P47226	Testin	5.43	Negative regulation of cell proliferation
P70362	Ubiquitin fusion degradation protein 1 homolog	4.10	Proteasome-mediated ubiquitin-dependent protein catabolic process
Q61074	Protein phosphatase 1G	3.63	Cell cycle arrest
Q9ERN0	Secretory carrier-associated membrane protein 2	3.56	Recycling carrier to the cell surface
Q9Z2M7	Phosphomannomutase 2	3.55	Fructose and mannose metabolism
P48771	Cytochrome c oxidase subunit 7A2, mitochondrial	3.39	Respiratory electron transport chain
Q8CI08	SLAIN motif-containing protein 2	3.29	Microtubule organization
P11730	Calcium/calmodulin-dependent protein kinase type II subunit gamma	3.02	Sarcoplasmic reticulum Ca^2+^ transport in skeletal muscle
Q9JHR7	Insulin-degrading enzyme	2.98	Degradation of insulin, glucagon and other polypeptides
Q8VCW4	Protein unc-93 homolog B1	2.97	Innate immune response
O08599	Syntaxin-binding protein 1	2.87	Unknown
P38060	Hydroxymethylglutaryl-CoA lyase, mitochondrial	2.74	Unknown
Q11136	Xaa-Pro dipeptidase	2.73	Collagen catabolic process
Q8K4M5	COMM domain-containing protein 1	2.68	Promotes ubiquitination of NF-kappa-B subunit RELA
O55012	Phosphatidylinositol-binding clathrin assembly protein	2.53	Endocytosis
P49442	Inositol polyphosphate 1-phosphatase	2.36	DNA synthesis inhibition
Q8BYK6	YTH domain family protein 3	2.36	mRNA splicing
O35682	Myeloid-associated differentiation marker	2.34	Strongly up-regulated as multipotent progenitor cells differentiate towards myeloid cells
Q4KML4	Costars family protein C6orf115 homolog	2.32	Unknown
Q61753	D-3-phosphoglycerate dehydrogenase	2.31	L-serine biosynthetic process
Q8VC28	Aldo-keto reductase family 1 member C13	2.31	Xenobiotic metabolic process
P97807	Fumarate hydratase, mitochondrial	2.28	Tricarboxylic acid cycle
Q8VBT0	Thioredoxin-related transmembrane protein 1	2.26	Cell redox homeostasis
Q3UX10	Tubulin alpha chain-like 3	2.26	Microtubule-based process
Q9JK38	Glucosamine 6-phosphate N-acetyltransferase	2.26	Glucosamine metabolic process
Q91V33	KH domain-containing, RNA-binding, signal transduction-associated protein 1	2.26	G2/M transition of mitotic cell cycle; alternative splicing

All listed proteins were identified in 2 samples based on two or more peptides identified for every protein; classified according to the fold change in expression based on global proteomic analysis. Selected functions of all listed proteins were assigned based on UniProtKB data base. ND- fold change in protein expression was not computed since the indicated proteins were not detected in Puro-treated cells.

**Table 2:** Proteins not found or expressed at lower level in MCPIP1-expressing MSCs when compared with Puro-treated MSCs.

Proteins not found in MCPIP1-overexpressing MSCs when compared with Puro			
*Accession number*:	*Protein name*	*Fold change*	*Selected function [Based on UniProtKB]*
P08592	Amyloid beta A4 protein	ND	Notch signaling and α-ATPase activity inhibition; couples to apoptosis-inducing pathways
Q9Z2G6	Protein sel-1 homolog 1	ND	Notch signaling negative regulation; misfolded ER proteins degradation
Q8C863	E3 ubiquitin-protein ligase Itchy	ND	Ubiquitinate CXCR4; Notch1 degradation controlling
Q4VSI4	Ubiquitin carboxyl-terminal hydrolase 7	ND	p53-dependent cell growth repressor and apoptosis inducer
Q9D0S9	Histidine triad nucleotide-binding protein 2, mitochondrial	ND	Apoptotic sensitizer
Q9CRA5	Golgi phosphoprotein 3	ND	Golgi membrane trafficking
Q3T1G7	Conserved oligomeric Golgi complex subunit 7	ND	Golgi function
P70280	Vesicle-associated membrane protein 7	ND	Transport vesicles targeting/ fusion
Q9ER41	Torsin-1B	ND	Secreted/membrane proteins folding
Q8JZK9	Hydroxymethylglutaryl-CoA synthase, cytoplasmic	ND	Fatty acid metabolism
Q6ZPR5	Sphingomyelin phosphodiesterase 4	ND	Catalyzer of phosphorylcholine and ceramide formation
Q8VEE4	Replication protein A 70 kDa DNA-binding subunit	ND	DNA replication and cellular response to DNA damage
P70583	Deoxyuridine 5'-triphosphate nucleotidohydrolase	ND	Nucleotide metabolism
Q61749	Translation initiation factor eIF-2B subunit delta	ND	Translation
Q8BG51	Mitochondrial Rho GTPase 1	ND	Mitochondrial trafficking
P05622	Platelet-derived growth factor receptor beta	ND	Cell proliferation, survival, differentiation, chemotaxis and migration
**Quantitatively less in MCPIP1-overexpressing MSCs when compared with Puro**			
Q80W54	CAAX prenyl protease 1 homolog	-5.17	Farnesylated proteins proteolytic processing
O35783	Calumenin	-5.09	Catalytic activity negative regulation
Q9JL26	Formin-like protein 1	-3.99	Cell morphology and cytoskeletal organization regulation
Q80TH2	Protein LAP2	-3.98	Nuclear envelope organisation
P53811	Phosphatidylinositol transfer protein beta isoform	-2.55	Catalyzer of PtdIns and phosphatidylcholine between membranes transfer
Q61655	ATP-dependent RNA helicase DDX19A	-3.34	Apoptotic process positive regulation; mRNA transport
Q8CCJ3	E3 UFM1-protein ligase 1	-3.28	NF-kappaB transcription factor activity negative regulation
P68433	Histone H3.1	-3.02	Nucleosome assembly
P17742	Peptidyl-prolyl cis-trans isomerase A	-2.89	Folding of proteins accelaration
Q6PGL7	WASH complex subunit FAM21	-2.78	Retrograde transport
O35864	COP9 signalosome complex subunit 5	-2.73	Unknown
P70452	Syntaxin-4	-2.65	Intracellular protein transport
P45878	Peptidyl-prolyl cis-trans isomerase FKBP2	-2.55	Peptidyl-proline modification
P84228	Histone H3.2	-2.49	Nucleosome assembly
Q9CQF9	Prenylcysteine oxidase	-2.43	Prenylated proteins degradation
Q9D071	MMS19 nucleotide excision repair protein homolog	-2.37	Unknown
Q91YP2	Neurolysin, mitochondrial	-2.31	Oligopeptides such as neurotensin, bradykinin and dynorphin A hydrolization
P15626	Glutathione S-transferase Mu 2	-2.31	Electrophilic compounds detoxification
Q9JIS8	Solute carrier family 12 member 4	-2.21	Potassium ion transport
Q9JIX8	Apoptotic chromatin condensation inducer in the nucleus	-2.03	Apoptotic process positive regulation

All listed proteins were identified in 2 samples based on two or more peptides identified for every protein; classified in accordance with the fold change in expression based on global proteomic analysis. Selected functions of the listed proteins were assigned based on UniProtKB data base. ND- fold change in protein expression was not computed since the indicated proteins were not detected in MCPIP1-overexpressing cells.

### MCPIP1 enhances the angiogenic potential of MSCs following stimulation accompanied by an increase in expression of proangiogenic factors

**Fig 3:**
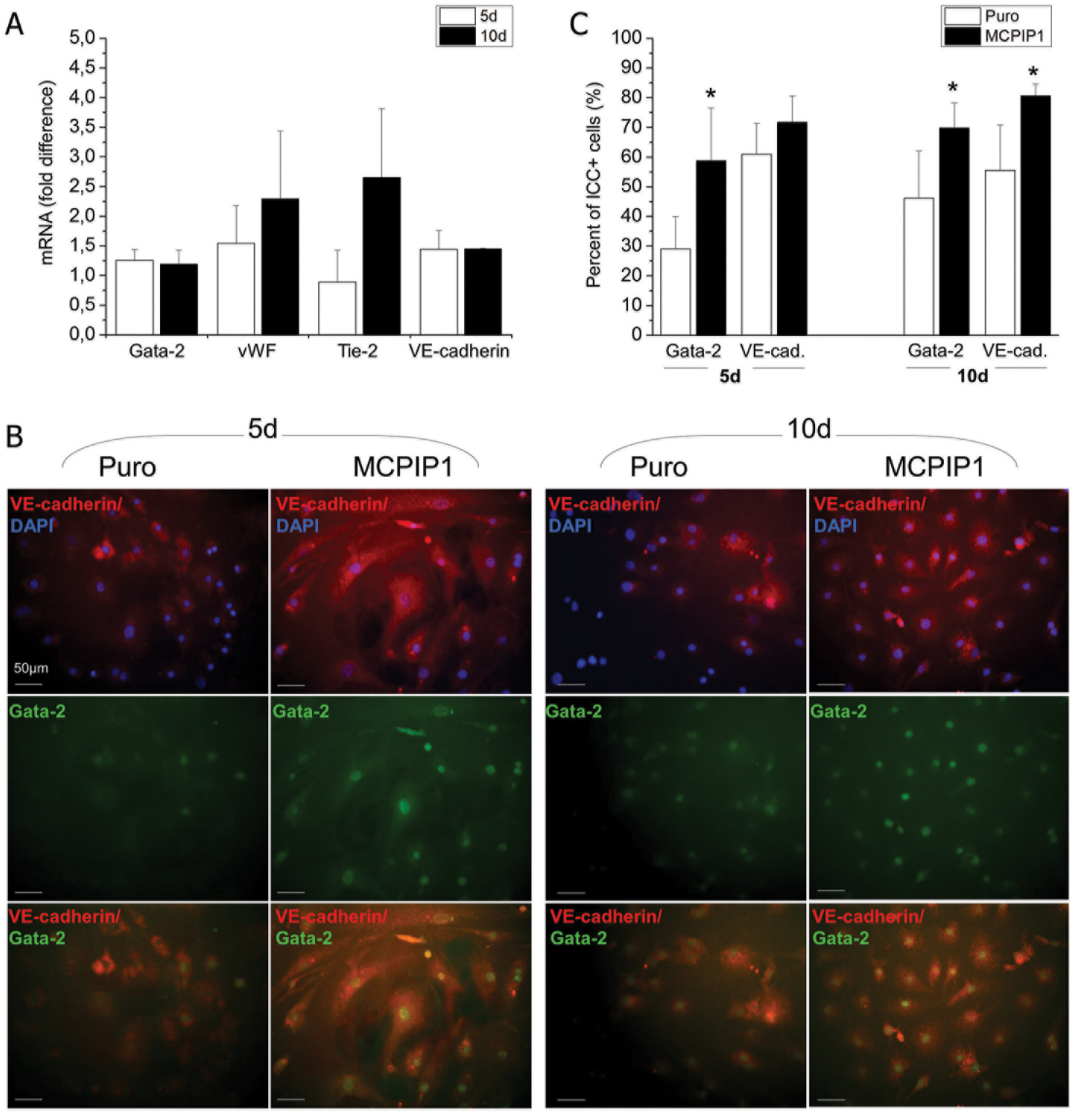
Expression of angiogenesis- related markers in MSCs after angiogenic differentiation. **(A)** Expression of mRNA for Gata-2, vWF, Tie-2 and VE-cadherin genes in MCPIP1- overexpressing MSCs after 5 and 10 days of angiogenic differentiation by real time RT-PCR. Fold change in mRNA concentration in MCPIP1- transduced MSCs was computed when compared with Puro-treated cells (calculated as 1). **(B)** Representative images of angiogenic marker expression assessed with immunocytochemistry in MCPIP1-overexpressing MSCs and Puro- treated MSCs differentiated into endothelial phenotype *in vitro*. MCPIP1-overexpressing MSCs and Puro were stained against intranuclear transcription factor Gata-2 (Alexa Fluor 488, green) and VE-cadherin (Alexa Fluor 546, red), whereas nuclei were co-stained with DAPI (blue). Cells were analyzed with Leica DM-IRE fluorescent microscope. Scale bars indicate 50μm. **(C)** Quantitative analysis of angiogenic differentiation of MCPIP1- overexpressing MSCs and Puro cells after 5 and 10d of culture. Graphs represent percentages of cells expressing the indicated angiogenic marker identified by immunohistochemisty within both MSC groups. All results are presented as means ± SD. Statistically significant differences (P<0.05) are shown when compared with Puro (*). Analysis based on three independent experiments. Puro—empty vector-treated MSCs; MCPIP1- MSCs overexpressing MCPIP1.

After establishing that MCPIP1-MSCs at 72h after transduction exhibited greater proangiogenic activity, we launched a long-term culture of these cells in proangiogenic medium (EGM-2MV) and assessed the angiogenic differentiation after 5d and 10d of culture (Fig 3). The expression of proangiogenic genes was investigated at mRNA level by real time RT-PCR and we found elevated expression of Gata-2, vWF and VE-cadherin after 5 days of angiogenic differentiation peaking at 10 days of culture in MCPIP1- MSCs when compared with Puro-treated cells (Fig 3A). Enhanced angiogenic capacity of MCPIP1-MSCs was confirmed in a direct differentiation assay followed by immunocytochemical staining for angiogenic proteins (Fig 3B and 3C). Quantitative analyses revealed the greatest number of cells with endothelial phenotype expressing intranuclear transcription factor Gata-2 and membrane VE-cadherin within MCPIP1-MSCs when compared with Puro cells (Fig 3C).

**Fig 4:**
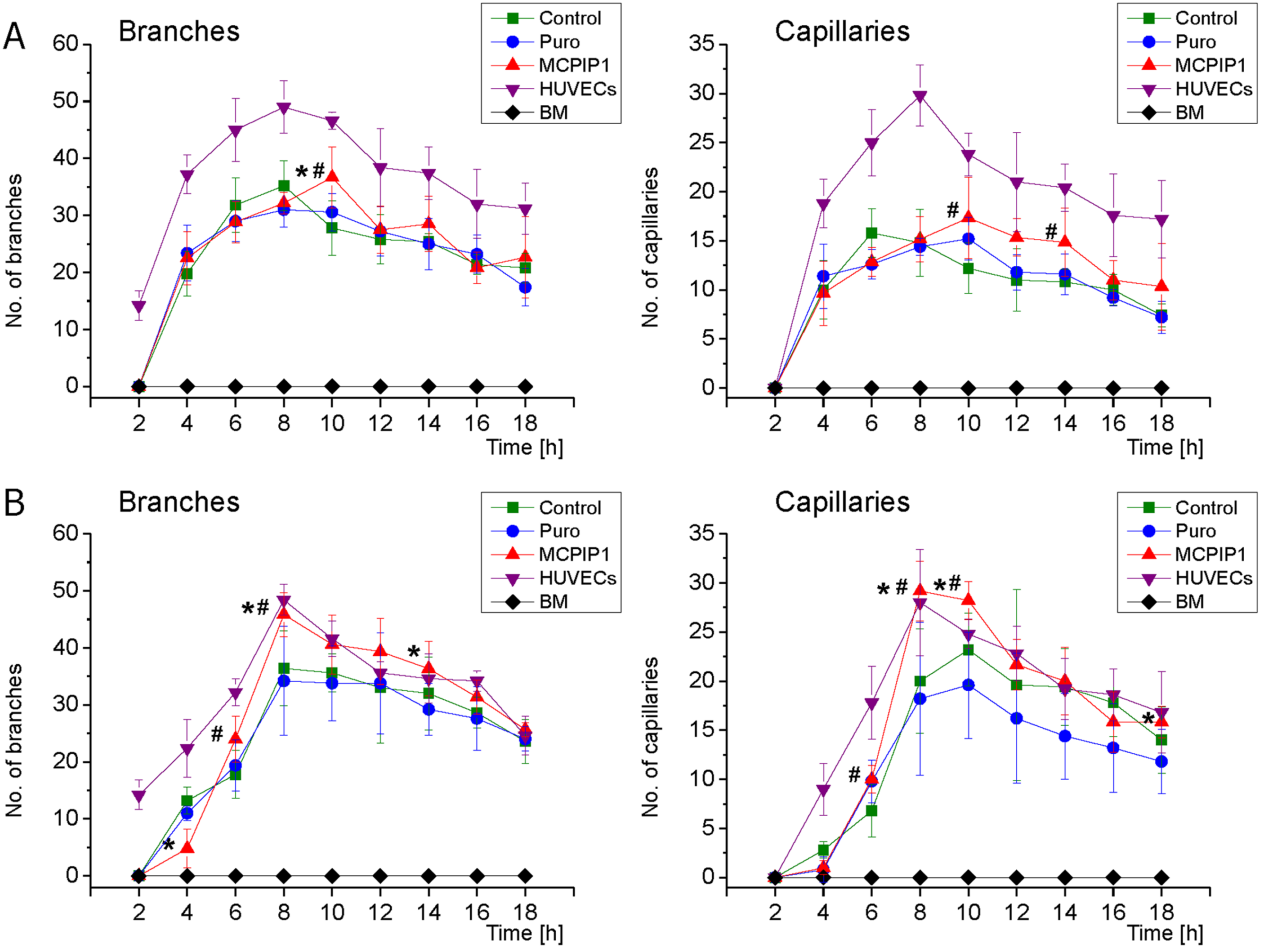
Functional angiogenic potential of MSCs following predifferentiation into endothelial cells by capillary-like formation assay. **(A)** Graphs represent quantitative assessment of branches (left graphs) and capillaries (right graphs) formed by MSCs differentiated in proangiogenic medium for 5 days. **(B)** Graphs show quantitative assessment of branches (left graphs) and capillaries (right graphs) formed by MSCs differentiated in proangiogenic medium for 10 days. HUVEC cells were used as positive control, while freshly isolated BM cells represent the negative control. Six randomly selected high-power field images were selected for quantification. Numbers of branches and capillaries formed by each cell fraction were computed based on microscopic images as shown in S2 Fig and the detailed results are presented in S2 Table. All results are presented as mean ± SD. Statistically significant differences (P<0.05) are shown when compared with Puro (*) and Control (#). Analysis based on three independent experiments. Control—untreated MSCs; Puro—empty vector-treated MSCs; MCPIP1- MSCs overexpressing MCPIP1.

Moreover, MCPIP1-overexpressing MSCs exhibited greater angiogenic activity in the functional Matrigel assay following pre-differentiation culture in proangiogenic medium (Fig 4). Quantitative analysis of tube formation *in vitro* (S2 Fig) showed a greater number of capillaries and branches within MSCs expressing MCPIP1 when compared with Puro-treated and untreated Control MSCs after 5 and 10 days of endothelial pre-differentiation (Fig 4A and 4B respectively; S2 Table).

**Fig 5:**
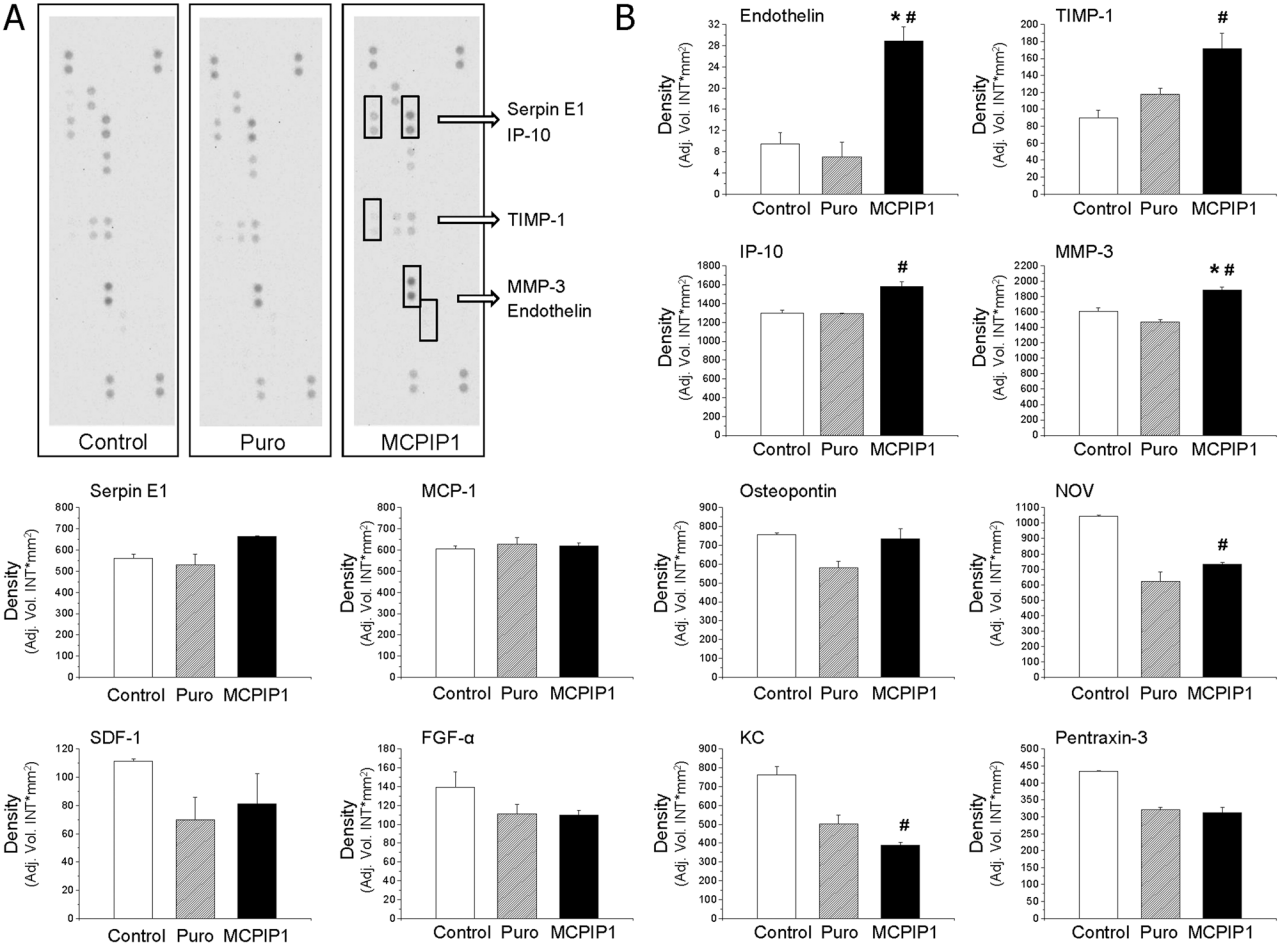
Semiquantitative analysis of angiogenesis-related proteins secreted by MSCs after 10 days of endothelial culture by Western blotting. **(A)** Representative nitrocellulose membranes incubated with conditioned culture medium harvested from cultures of all three experimental groups of MSC (MCPIP1-overexpressing MSCs, empty vector- treated (Puro) MSCs and untreated (Control) MSCs). Pairs of duplicate spots represent each angiogenesis- related protein. Pair of duplicate spots with upregulated expression when compared with control cells were included in brackets. **(B)** Semiquantitative assessment of selected protein concentrations based on pixel density analysis with Quantity One software. All results are presented as means ± SD. Statistically significant differences (P<0.05) are shown when compared with Puro (*) and Control (#). The analysis was conducted using a mixture of conditioned media collected under cells prepared from three independent experiments. Control—untreated MSCs; Puro—empty vector-treated MSCs; MCPIP1- MSCs overexpressing MCPIP1.

To determine whether MCPIP1-overexpressing MSCs are able to secrete any protein involved in angiogenesis that may stimulate neighboring cells in a paracrine manner, we compared the secretome of MSCs overexpressing MCPIP1 and control MSCs by using Western immunoblotting as well as quantitative analysis with the Luminex platform (Fig 5 and S3 Fig). Based on our previous experimental data indicating that MCPIP1 up-regulates angiogenesis-related genes and promotes capillary-like tube formation especially after 10 days following endothelial stimulation, we used conditioned cell culture medium derived from MCPIP1-MSCs and control MSCs at this time point. We found that secretion of endothelin, a tissue inhibitor of metalloproteinase-1 (TIMP-1), Serpin E1, IFN-γ inducible- protein-10 (IP-10), matrix metalloproteinase-3 (MMP-3), stromal cell-derived factor 1 (SDF-1), osteopontin and insulin-like growth factor-binding protein 9 were significantly upregulated in MCPIP1-overexpressing MSCs when compared with Puro cells (Fig 5). Next, we employed the quantitative Luminex-based assay where we tested the secretion of selected proangiogenic proteins by MCPIP1-overexpressing MSCs and controls at day 0, 5 and 10 following endothelial differentiation (S3 Fig). We detected elevated concentration of endoglin in MCPIP1-overexpressing MSCs when compared with Puro-treated cells, especially after 10 days of differentiation (S3 Fig). Similar to previous results, the concentration of endothelin increased slightly after 5 and 10 days of pre-differentiation. Interestingly, in case of VEGF, we observed a decreased level of this factor along the differentiation culture in all groups. In contrast, the concentration of SDF-1 increased after 10 days of stimulation (Fig 5 and S3 Fig).

Importantly, with both assays, we did not detect any measurable level of IL-1β or other inflammatory cytokines or chemokines produced by MCPIP1-overexpressing MSCs (data not shown). These data confirm the anti-inflammatory properties of MCPIP1 also in MSCs that have already been reported for other cell types [21, 23]. Finally, we detected decreased concentration of MCP-1 released by MCPIP1-overexpressing MSCs when compared with Puro-treated and untreated MSCs that remained consistent during differentiation culture (S3 Fig). This may suggest a negative feedback loop of MCP-1 regulation by MCPIP1 in MSCs.

### MCPIP1 promotes cardiomyogenic differentiation of MSCs

**Fig 6:**
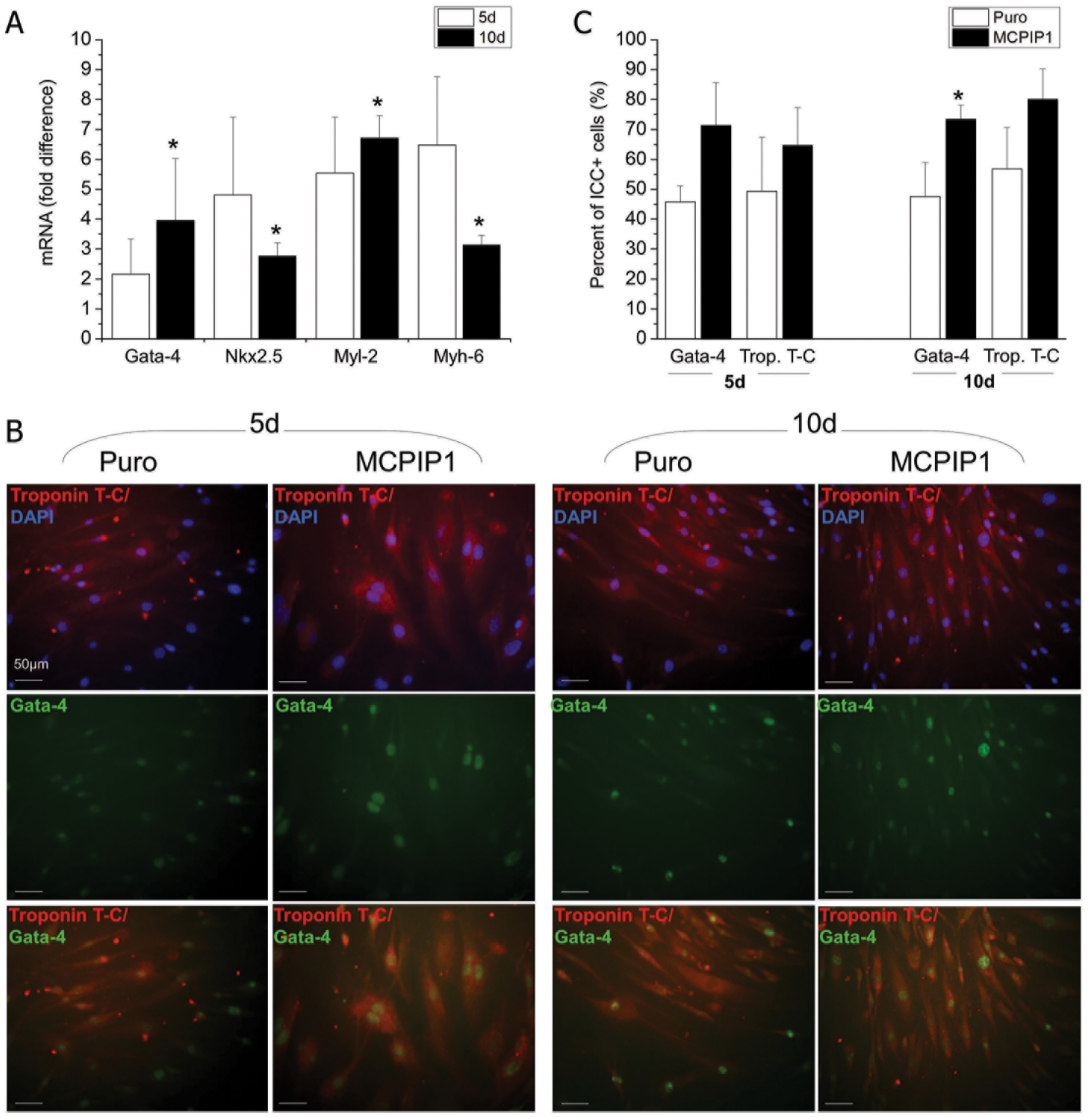
Expression of cardiac markers in MSCs after cardiomyogenic differentiation. **(A)** Expression of mRNA for Nkx2.5, Gata-4, Myl-2 and Myh-6 genes after 5 and 10 days of cardiac differentiation by real time RT-PCR. Fold change in mRNA concentration in MCPIP1- transduced MSCs was computed when compared with Puro-treated cells (calculated as 1). **(B)** Representative images of cardiac markers expression assessed with immunocytochemistry in MCPIP1-overexpressing MSCs and Puro- treated MSCs differentiated into cardiac phenotype *in vitro*. MCPIP1-overexpressing MSCs and Puro were stained against intranuclear transcription factor Gata-4 (Alexa Fluor 488, green) and Troponin T-C (Alexa Fluor 546, red), whereas nuclei were co-stained with DAPI (blue). Cells were analyzed with a Leica DM-IRE fluorescent microscope. Scale bars indicate 50μm. **(C)** Quantitative analysis of cardiomyogenic differentiation of MCPIP1-overexpressing MSCs and Puro cells after 5 and 10d of culture. Graphs represent percentages of cells expressing the indicated cardiac marker identified by immunocytochemisty within both MSC groups. All results are presented as means ± SD. Statistically significant differences (P<0.05) are shown when compared with Puro (*). Analysis based on three independent experiments. Puro—empty vector-treated MSCs; MCPIP1- MSCs overexpressing MCPIP1.

To establish the impact of MCPIP1 on cardiac differentiation, MCPIP1-MSCs and control cells were differentiated into cardiomyocytes *in vitro* as previously described [32]. We analyzed mRNA expression for cardiac markers such as Gata-4, Nkx2.5, Myl-2 and Myh-6 after 5 and 10 days of differentiation induction (Fig 6A). We found that the expression of these genes was markedly elevated after 5 and 10 days of cardiomyogenic differentiation induction in MCPIP1-overexpressing MSCs when compared with Puro-treated cells (Fig 6A).

**Fig 7:**
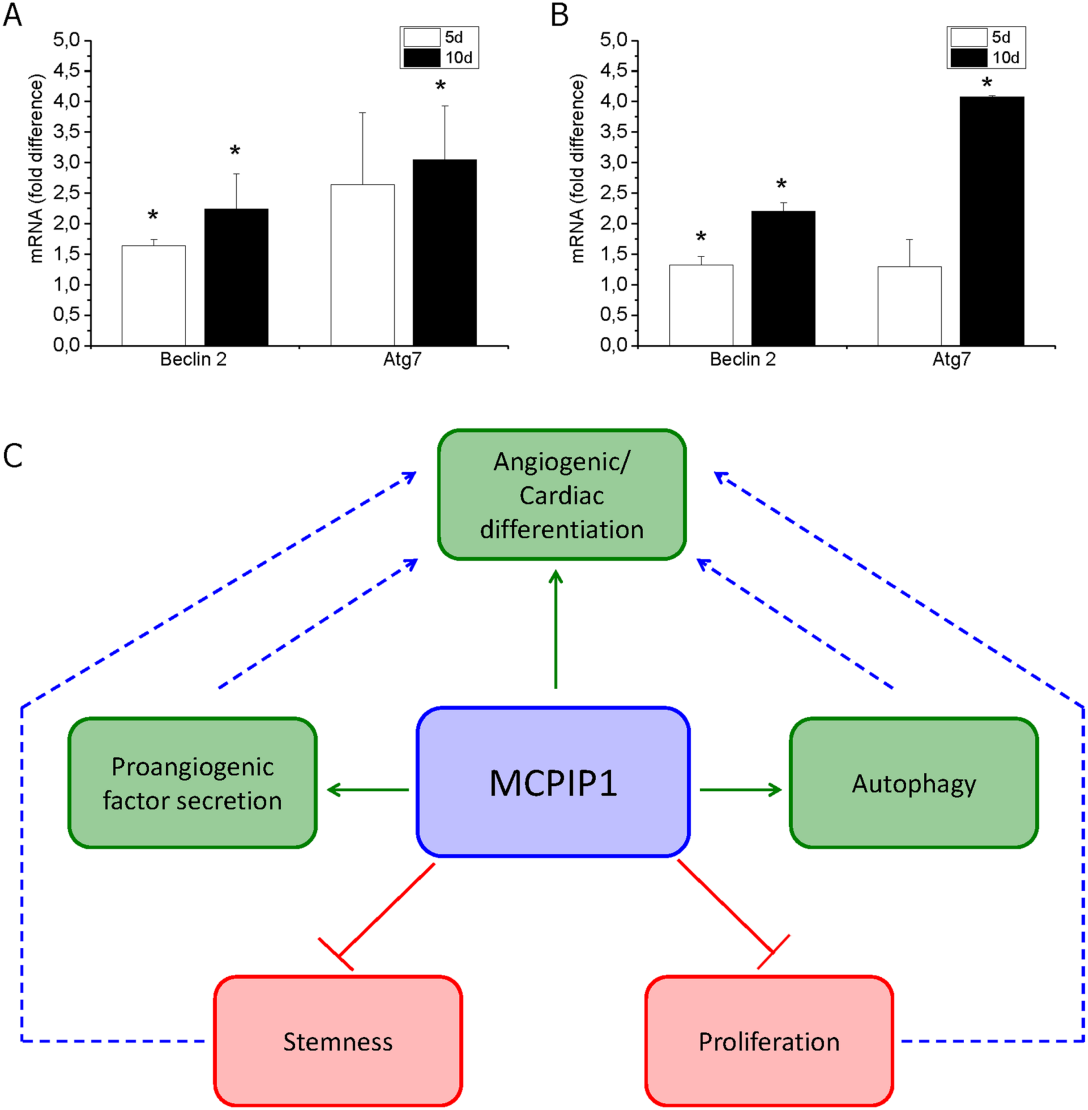
Expression of autophagy- related genes in MSCs during differentiation. Expression of mRNA for Beclin-2 and Atg7 in MCPIP1- overexpressing MSCs after 5 and 10 days of cardiomyogenic **(A)** and angiogenic **(B)** differentiation by real time RT-PCR. Fold change in mRNA concentration in MCPIP1- transduced MSCs was computed when compared with Puro-treated cells (calculated as 1). All results are presented as means ± SD. Statistically significant differences (P<0.05) are shown when compared with Puro (*). Analysis based on three independent experiments. **(C)** Potential functional network impacted by MCPIP1 in MSC cells. MCPIP1 increases angiogenic and cardiac differentiation capacity of MSCs which is accompanied with decrease in expression of early stem cell-related genes and proliferation rate as well as with increase in expression of tissue commitment- related genes and several proteins involved in the differentiation processes. Moreover, the increase in differentiation activity of MSCs may be accompanied with intracellular reorganization involving process of autophagy.

Enhanced cardiomyogenic capacity of MSCs overexpressing MCPIP1 was confirmed by immunocytochemical staining for typical cardiac proteins following differentiation in cardiac medium *in vitro* (Fig 6B and 6C). Quantitative assessment of differentiating MSCs (after 5 and 10 days of culture) revealed greater numbers of cells with cardiac phenotype expressing intranuclear cardiac transcription factor Gata-4 and cytoplasmic structural protein troponin T-C within MCPIP1-MSCs when compared with Puro cells (Fig 6C). Interestingly, we found increase in expression of autophagy related genes in MCPIP1-MSCs undergoing cardiac or angiogenic differentiation (Fig 7A and 7B).

Our findings indicate that MCPIP1 protein enhances not only angiogenic, but also cardiomyogenic differentiation of MSCs. Interestingly, our gene expression and proteomic studies suggest that such processes may be accompanied with intracellular reorganization via autophagy, which may represent new interesting subject for future studies and needs to be further investigated (Fig 7C).

## Discussion

According to annual reports of the World Health Organization, ischemic heart disease (IHD) is one of the leading causes of death worldwide [33]. IHD may lead to acute ischemic events such as myocardial infarction (MI) resulting in massive cardiomyocyte death due to apoptosis and necrosis [34]. The profound inflammatory reaction that follows ischemia contributes progression of ventricular remodeling and eventually dysfunction, heart failure and higher mortality [35]. Therapeutic approaches have therefore been aimed at inhibiting the adverse consequences of myocyte loss and tissue remodeling. These have included both pharmacological methods and cell-based regenerative strategies with stem cells [36–38].

Bone marrow MSCs, a subset of adherent cells with multipotent differentiation capacity, are able to differentiate into several cell types, including cardiomyocytes and endothelial cells [39, 40]. Due to this broad differentiation potential, low immunogenicity, and anti-inflammatory activities, MSCs have been widely employed for myocardial salvage, repair, and regeneration following ischemic injury [41–44].

In this study, BM-derived MSCs were genetically modified to overexpress MCPIP1, a protein exhibiting a strong silencing potential of the inflammatory response and modulation of gene expression by RNase activity [21, 23, 24]. MCPIP1 has also recently been implicated in adipogenesis [28] and angiogenesis [25]. Although, a role of MCPIP1 in cardiomyocyte survival was reported [26, 45], the role of this protein in cardiomyogenesis from stem cells has never been examined.

In view of the role of MCPIP1 in cell survival and initiation of apoptosis [25, 46, 47], we evaluated the activation of proapoptotic caspase-3 and -7 as well as the extent of cells undergoing apoptosis and necrosis. By employing several independent assays, we showed that MCPIP1 overexpression did not negatively impact the viability of MSCs. Moreover, we observed normal metabolic activity based on measurement of total ATP concentration in such cells. These results indicate greater resistance of stem cells such as BM-derived MSCs against activation of apoptosis via MCPIP1 when compared with other more mature cells such as primary HUVECs and cardiomyocytes previously examined by other groups [25, 46, 47].

Interestingly, other studies have reported a beneficial effect of cardiac MCPIP1 expression on myocardial injury. Using a transgenic mouse model with myocardial MCPIP1 expression (under α-MHC promoter), Niu et al. reported significant attenuation of inflammation-related cardiac dysfunction [26, 45]. The authors observed decreased expression of inflammatory cytokines and iNOS as well as caspase 3/7 activity and apoptosis in MCPIP1-expressing myocytes following treatment with LPS when compared with wild type animals [26, 45]. These results were confirmed by other investigators reporting lower levels of inflammatory cytokines (e.g. TNF-α, IL-1β) along with higher levels of anti-inflammatory cytokines (e.g. IL-10) in myocardium of animals with MCPIP1 expression following LPS treatment [48–50]. This phenomenon may be related to RNase activity of MCPIP1 due to the presence of the PilT N-terminus domain which has been shown to promote degradation of mRNA for inflammatory cytokines such as IL-6 and IL-1 as well as select pre-miRNAs that regulate other factors involved in inflammation [21, 23, 24]. Moreover, Morimoto et al. have demonstrated a cardioprotective effect of MCP-1 (acting via stimulation of MCPIP1 expression) on myocardium following ischemia/ reperfusion (I/R) injury in mice [51, 52]. Thus, the findings suggest that MSCs overexpressing MCPIP1 may have an anti-apoptotic and anti-inflammatory role on myocardium following transplantation into ischemic heart tissue.

Moreover, MCPIP1 may also be involved in deubiquitination of TRAF family proteins which are known to mediate inflammatory response [53]. Interestingly, our proteomic analysis of MSCs from all three experimental groups revealed expression of ubiquitin-conjugating enzyme E2 H involved in protein ubiquitination and ubiquitin fusion degradation protein 1 responsible for proteasome-mediated protein catabolic process in MCPIP1-overexpressing MSCs, but not in control MSCs. Thus, these data suggest that MCPIP1 expressed in MSCs may indirectly enhance ubiquitination of selected proteins resulting in their degradation. This phenomenon may also potentially be involved in inhibition of inflammatory cytokine production by MCPIP1-MSCs; however, this needs to be further evaluated. Overall, these data again indicate that MCPIP-1 may exert anti-inflammatory and anti-apoptotic properties in MSCs.

Interestingly, we found that although MCPIP1 did not notably affect morphology or antigenic phenotype of MSCs, it significantly impaired proliferation of MSCs expressing MCPIP1 when compared with Puro vector-treated cells. Consistently, global proteomic analysis revealed that MCPIP1-MSCs expressed greater levels of several proteins involved in negative regulation of cell proliferation and cell cycle arrest, including testin and protein phosphatase 1G. It is generally known that a decrease in cell proliferation may often be accompanied by induction of cell differentiation [54, 55]. Thus, we expected that the effect of MCPIP1 on MSC proliferation may be related to greater differentiation status of these cells confirmed by further experiments.

It has been reported that MCPIP1 may enhance the angiogenic potential of HUVEC cells, which represent mature cells, and BM-derived monocytic cells [25, 56]. Using Matrigel assay, Niu and colleagues have shown that HUVECs stimulated with MCP-1 show greater angiogenic potential. Importantly, knockdown of MCPIP1 in HUVECs resulted in suppression of VEGF and hypoxia-inducible factor-1α (HIF-1α) genes, the angiogenesis- related factors induced by MCP-1 [25]. Similarly, angiogenic differentiation of human BM-derived monocytic cells (BMNCs) with MCPIP1 expression and enhanced neovascularization after transplantation into ischemic tissues *in vivo* has also been reported [56]. However, the impact of MCPIP1 on the angiogenic capacity of stem cells such as MSCs has never been studied.

Our results show, for the first time, that MCPIP1 may enhance angiogenic differentiation of adult stem cells such as MSCs *in vitro*. Our results also reveal significant upregulation of endothelial transcription factors and structural proteins, including Gata-2 and VE-cadherin at both mRNA and protein levels in MSCs expressing MCPIP1 following culture in proangiogenic medium. Moreover, these data also establish that MCPIP1 enhances formation of capillary-like structures in Matrigel assay after proangiogenic stimulation. Interestingly, the greater angiogenic capacity of MCPIP1-MSCs was accompanied by decreased expression of early stem cell-related genes such as Oct-4, Nanog and Sox2. Our data indicate enhanced angiogenic capacity of MSCs expressing MCPIP1 when compared with control cells confirming proangiogenic activity of MCPIP1 not only in mature cells but also in stem cells.

In the present study we also report, for the first time, that MCPIP1 expression enhances differentiation of BM-derived MSCs into cardiomyocytes *in vitro*. We found that MCPIP1 promoted the acquisition of a cardiac phenotype by MSCs, evidenced by upregulation of cardiomyogenesis-related transcription factors, such as Gata-4 and Nkx2.5, as well as structural proteins, including Myh-6 and Myl-2. Furthermore, we detected a greater number of cells expressing Gata-4 and troponin T-C at the protein level in MCPIP1-MSCs following 10 days of culture in cardiomyogenic medium. Our findings therefore support a role of MCPIP1 in generation of cardiomyocytes stem cells. These data also provide the rationale to develop novel strategies to transplant MSCs overexpressing MCPIP-1 into ischemic myocardium to enhance cardiac and vascular regeneration.

Current evidence from *in vitro* and *in vivo* studies strongly indicate that major mechanisms of stem cell-mediated neovascularization include not only effective stem cell retention and vascular differentiation in the infarcted heart, but also secretion of several factors impacting endogenous cells in a paracrine manner [57, 58]. Interestingly, we found that MCPIP1-overexpressing MSCs secrete higher levels of several proteins involved in angiogenesis including endoglin, endothelin, TIMP-1, serpin E1, IP-10 and MMP-3, when compared with controls. Furthermore, we observed a higher level of chemokine SDF-1 secreted by MSCs expressing MCPIP1 when compared with control cells. SDF-1 has been shown to play a pivotal role in directing CXCR4+ stem cells toward the site of injury, an essential step for cardiac repair [59–61]. Our data suggest that production of SDF-1 by transplanted MCPIP1-MSCs may enhance homing of other endogenous stem cells to the infarcted heart and promote regeneration. Aside from serving as a homing factor, SDF-1 has also been shown to be a proangiogenic factor [62, 63], and as such may increase new vessel formation when released by transplanted MCPIP1-MSCs.

Interestingly, it has been recently demonstrated that forced expression of MCPIP1 may induce autophagy in HUVECs finally leading to improvement of angiogenic potential [64]. The phenomenon may be explained by the necessary rebuilding of cell contents during differentiation via elimination of several proteins and organelles associated with metabolic changes [65–67]. Our global proteomic analysis showed increased expression of autophagy regulators, such as CDGSH iron-sulfur domain-containing protein 2 exclusively in MSCs expressing MCPIP1. Importantly, the expression of genes related to autophagy such as beclin 2 and Atg7 was greatly increased in MCPIP1-MSCs during their angiogenic and cardiac differentiation. Moreover, we found several proteins potentially involved in the intracellular cytoskeleton and membrane reorganization to be expressed at a higher level in MCPIP1-MSCs, including Ras-related protein Rab-11B, tubulin alpha chain-like 3 or SLAIN motif-containing protein involved in microtubule organization. Elevated expression of proteins involved in intracellular membrane trafficking and recruiting effector molecules critical for vesicle traffic along actin- or microtubule-based cytoskeletal structures, such as secretory carrier-associated proteins or Rab proteins was also reported [68]. The data may indicate that MSCs expressing MCPIP1 contain a set of molecules participating in intracellular component remodeling and vesicular transport facilitating differentiation processes in these SCs. Thus, the observed greater angiogenic and cardiac differentiation potential of MCPIP1-MSCs may be preceded by autophagy related to MCPIP1 expression and intracellular reorganization. However, this interesting phenomenon warrants further investigation.

In summary, overexpression of MCPIP1 reduces the expression of pluripotency associated markers, and increases angiogenic and cardiomyogenic potential of MSCs. MCPIP1 does not notably impact MSC viability, metabolic activity, morphology, and antigenic profile, yet reduced the proliferation rate. MCPIP1-overexpressing MSCs express several proteins potentially involved in angiogenesis, autophagy and processes accompanying cell differentiation (Fig 7C). These results strongly indicate the beneficial impact of MCPIP1 protein on MSC features which may be utilized for potential clinical applications.

## Supporting Information

S1 Appendix Extended Materials and Methods. (DOC)

S1 Fig Transduction efficiency and MCPIP1 overexpression in murine BM- derived MSCs at 72h post transduction. Morphology of the entire population of BM- derived MSCs following transduction with a vector carrying GFP by fluorescence (FL) microscopy. BF—Brightfield image, green FL—GFP- expressing MSCs, combo—combined image. Scale bars: 100 μm. (B) Representative histograms visualizing average values of transduction efficiency with pMX-based retroviral vector carrying GFP in the whole MSC population when compared with non-transduced Control MSCs. (C) Quantitative analysis of mRNA concentration for MCPIP1 at 48h and 72h following retroviral transduction. Black bars represent MSCs transduced with vector carrying MCPIP1 sequence, while hatched and white bars show MSCs transduced with empty vector (Puro) or untreated (Control) MSCs, respectively. The expression of MCPIP1 in Puro-treated cells is set as 1. (D) MCPIP1 overexpression in murine BM- derived MSCs. Upper panel: Representative Western blotting analysis of MCPIP1 protein expression at 48 and 72h following transduction. Actin was used as endogenous control. Lower panel: Semiquantitative assessment of MCPIP1 protein and actin concentrations based on pixel density analysis with Quantity One software. (E) Viability of MCPIP1-overexpressing MSCs when compared with Puro-treated cells (set as 1) at 48 and 72h following transduction by MTT assay. (F) Metabolic activity of MCPIP1-overexpressing MSCs when compared with Puro-treated cells (set as 1) assessed by ATP concentration measurement. All results are presented as mean ± SD. Statistically significant differences (P<0.05) are shown in comparison with Puro (*) and untreated Control (#) cells. Analysis based on three independent experiments. Control—untreated MSCs; Puro—empty vector-treated MSCs; MCPIP1- MSCs overexpressing MCPIP1.
(TIF)

S2 Fig Strategy for semi-quantitative analysis of angiogenic potential determined by capillary-like formation assay. Six representative brightfield images of high-power fields (objective magnification 4x) were randomly selected and taken at every experimental timepoint for quantitative assessment. (A) Total number of capillaries were counted as shown by circles. (B) Total number of branches were assessed as shown by crosses. Average mean and SD were computed for every experimental timepoint. Control—untreated MSCs; Puro—empty vector-treated MSCs; MCPIP1- MSCs overexpressing MCPIP1.
(TIF)

S3 Fig Quantitative analysis of angiogenesis-related proteins secreted by MSCs after 5 and 10 days of proangiogenic stimulation. Concentration of analytes was evaluated in cell culture supernatants harvested from cultures of all three experimental groups of MSC (MCPIP1-overexpressing MSCs, empty vector- treated (Puro) MSCs and untreated (Control) MSCs) with Luminex xMAP technology using Mouse Angiogenesis/ Growth Factor Magnetic Bead Panel. Control—untreated MSCs; Puro—empty vector-treated MSCs; MCPIP1- MSCs overexpressing MCPIP1.
(TIF)

S1 Table Quantitative analysis of number of branches and capillaries formed by MSCs in capillary-like formation assay—number of branches and capillaries calculated per field formed by non-differentiated MSC groups (at 72h following transduction). (DOC)

S2 Table Quantitative analysis of number of branches and capillaries formed by MSCs in capillary-like formation assay—number of branches and capillaries formed by MSC groups after 5 and 10 days of endothelial stimulation. (DOC)
