# Supplementary material for: Monocyte Chemoattractant Protein-Induced Protein 1 (MCPIP1) Enhances Angiogenic and Cardiomyogenic Potential of Murine Bone Marrow-Derived Mesenchymal Stem Cells
Source: PLoS One. 2015 Jul 27;10(7):e0133746. doi: 10.1371/journal.pone.0133746 (PMC4516329; doi:10.1371/journal.pone.0133746)
Supplement: S1 Appendix — (DOC) [file pone.0133746.s001.doc]

**S1 APPENDIX**

**EXTENDED MATERIALS AND METHODS**

**Animals**

4-6 week old C57Bl/6 mice were obtained from animal facility from University Children’s Hospital in Krakow (Poland). All procedures and experiments involving animal material were performed in accordance with the national and European legislation following approval by the First Local Ethical Committee on Animal Testing at the Jagiellonian University in Krakow (approval number: 31/2012).

**Isolation and culture of BM-derived MSCs**

Bone marrow (BM)- derived cells were harvested from tibias and femurs by flushing the bones with Dulbecco’s Modified Eagle’s Medium/Nutrient Mixture F-12 Ham (DMEM/F12; Sigma-Aldrich). BM-derived cells were centrifuged (300xg, 5 min.), re-suspended in DMEM/F12 supplemented with 10% fetal bovine serum (FBS; Sigma-Aldrich), 100U/ml of penicillin and 0.1mg/ml of streptomycin (Gibco, Life Technologies) and seeded into Primaria culture flask (BD Falcon) at the density of 25x10^6^ nucleated cells/ 75cm^2^. Flushed bones were fragmented and enzymatically digested with collagenases type I and II (at the concentration of each 1 mg/ml; Sigma Aldrich) for 1.5h at 37^o^C, and subsequently washed and added equally to culture flasks containing flushed BM cells. Cells were cultured in a humidified atmosphere at 5% CO2 and 37˚C for 72h, and therefore non-adherent cells and bone fragments were removed form flasks. The culture medium was changed twice a week and the passage was performed with 0.25% trypsin/ EDTA (Gibco, Life Technologies) when confluence of cells reached close to 90%.

**Retroviral generation**

Retroviral vectors harboring following plasmids were used in the study: pMX-MCPIP1 coding for wild-type MCPIP1, pMX-GFP (control plasmid containing insert with enhanced green fluorescence protein (eGFP)), and pMX-Puro (empty plasmid, devoid of any insert). Coding sequence of human MCPIP1 was cloned into the empty vector using BamHI and NotI restriction sites. Retrovirus packaging was performed in modified HEK293 (Phoenix Amphotropic) as described earlier . After 48h following transfection supernatants containing vesicular stomatitis virus glycoprotein (VSV-G)- pseudotyped retroviral particles were filtered and frozen for experiments. To keep reproducible conditions between experiments, each harvest of retrovirus was examined in respect of a relative titer of the pMX-MCPIP1, pMX-eGFP and pMX-Puro vectors. For this purpose, RNA was analytically isolated from 300ml of the supernatant. After enzymatic digestion of DNA contamination the RNA was subjected to reverse transcription and real-time PCR using the MMLV-Reverse Transcriptase (Promega) and Sybr Green-based master mix B (A&A Biotechnology) was performed. Primers were complementary to the MMLV psi packaging element (F: GAACTGACGAGTTCGGAACAC; R: CCAGAACCACATATCCCTCCTCTA) lying within the backbone of pMX-expressing vectors. Primers allowed for cDNA synthesis and for the further PCR amplification. Then, biological titer was assessed by flow cytometry analysis (LRS II, Becton Dickinson) at HeLa cells transiently transduced with pMX-eGFP.

**Retroviral transduction**

To obtain MCPIP1 overexpressing murine MSCs, retrovirus- mediated gene transfer based on *Kitamura et al.* pMX-Puro system was performed . Murine BM- derived MSCs on passage 3 to 4 were transduced with retroviral vectors: pMX-MCPIP1, pMX-GFP and pMX-Puro. Each retroviral vector was added to 0.5x10^6^ MSCs in the presence of 4 μg/ml polybrene (Merck Millipore) in complete growth medium (MOI=7). Cells were subsequently seeded and cultured on 0.1% gelatin-coated (Sigma-Aldrich) 10 cm in diameter culture dishes (BD Falcon). After 24h, medium was changed. In order to increase the population of MCPIP1-overexpressing cells, transduction was repeated after 48h. Cells were subjected to analysis at 48 and 72h post double transduction. Potential impact of MSCs transduction with pMX-MCPIP1 vector as well empty vector (pMX-Puro) on morphology of recipient cells was investigated by phase-contrast microscopy (Leica DMI 4000B fluorescence microscope).

**Viability, proliferation and metabolic activity assessment**

The viability and metabolic status of MSCs were measured by MTT assay and analysis of ATP content, respectively. The test were performed in MSCs at 48 and 72h post second transduction.

For MTT assay cells were seeded on transparent 96-well plates (BD Falcon) to reach 90% of confluence. Then MTT (3-(4,5- dimethylthiazol-2-yl)-2,5-diphenyl-tetrazolium bromide) (Sigma-Aldrich) was added at a final concentration of 500 ng/ml for 4h. The medium was subsequently removed and MTT crystals were dissolved in acidic isopropanol (40 mM HCl; POCh) by shaking at 300 rpm for 30 minutes. The absorbance was measured with Infinite 200 microplate reader (Tecan Group Ltd.) at a wavelength of 570 nm with background subtraction at 650 nm.

For ATP concentration measurement, MSCs were seeded on 96-well white plates to obtain 90% of confluence in each time point and ATP Lite Luminescence assay kit was performed according to manufacturer’s instructions (Perkin Elmer). Luminescence was measured using SpectraFluor Plus (Tecan Group Ltd) microplate reader**.**

The relative proliferation ratio of MSCs was evaluated at 72h after second transduction. MSCs were counted by employing Countess II Automated Cell Counter (Life Technologies).

**Western blotting**

Expression of MCPIP1 on protein level was detected by western blotting analysis . Total MSCs lysates were prepared using RIPA buffer supplemented with protease inhibitors (Sigma-Aldrich) at each time points (48 and 72h post second transduction). Proteins included in samples (50μg of total protein) were further separated on 10% sodium dodecyl sulfate polyacrylamide gel electrophoresis (SDS-PAGE) and electrotransferred to PVDF membrane (Merck Millipore). Membranes were then blocked in Tris-buffered saline (150 mm NaCl, 20 mm Tris pH 7.6) containing 0.1% Tween 20 and 3% milk for 1h at RT. The presence of MCPIP1 was assessed using primary rabbit anti-MCPIP1 antibody (own production; 1:2000) . As a loading control, primary mouse anti-actin antibody (Sigma; 1:1000) was used. Membranes were incubated with primary antibodies at 4°C overnight. Membranes were washed and specific bands indicating specific proteins were detected with HRP-conjugated anti-rabbit secondary antibody (Cell Signalling; 1:6000) or HRP-conjugated anti-mouse secondary antibody (Sigma-Aldrich; 1:2000). The bands were visualized with an enhanced chemiluminescence reagent Luminata Crescendo (Merck Millipore) and images were collected by ChemiDoc XRS chemiluminescence detector (Bio-Rad). Densitometry analysis was performed for each time separately in respect to pMX-Puro-treated cells.

**Antigenic phenotyping by flow cytometry**

The phenotype of MSCs was evaluated at 72h after second transduction representing the timpoint with the highest expression of MCPIP1 following the infection. Thus, MSCs overexpressing MCPIP1 as well as controls – pMX-Puro-treated and untreated cells (Control) were immunolabelled with the following monoclonal antibodies: anti-CD45 (APC-Cy7 or FITC, clone: 30-F11, BD Bioscence), anti-Sca-1 (PE, clone: E13-161.7, BD Bioscence), anti-CD105 (PE/Cy7, clone: MJ7/18, BioLegend), anti- CD90.2 (APC, clone: 30-H12, BioLegend). Staining was performed according to manufacturer’s protocols for 30 min at 4^o^C. Cells were subsequently washed in phosphate buffered saline (DPBS; w/o Ca^2+^, Mg^2+^; HyClone), re-suspended in DMEM/F12 containing 2% FBS and collected by LSR II flow cytometer (Becton Dickinson). The percentage of the following cell subpopulations within CD45-negative MSC compartment (representing non-hematopoietic cells) were analyzed with FACS Diva software (Becton Dickinson): CD105+/Sca-1+, CD105+/Sca-1-, CD105-/Sca-1+, CD105-/Sca-1-, CD90+/Sca-1+, CD90+/Sca-1-, CD90-/Sca-1+, CD90-/ Sca-1-, CD105+/CD90+, CD105+/CD90-, CD105-/CD90+, CD105-/CD90-. The content of all fractions was analyzed in both MCPIP1 overexpressing and control MSCs (pMX-Puro-treated and untreated cells).

**Necrosis and apoptosis detection**

Assessment of necrosis and different apoptosis stages was performed by employing PE-conjugated Annexin V Apoptosis Detection Kit (BD Biosciences) with flow cytometry. MSCs overexpressing MCPIP1 as well as controls – pMX-Puro-treated and untreated cells (Control) were analyzed at 72h following transduction. Thus, the cells were washed twice with cold DPBS and re-suspended in 1X Binding Buffer according to manufacturer’s protocol. 1x10^5^ cells were labeled with PE- conjugated Annexin V (AnnV) and 7-aminoactinomycin (7-AAD) for 15 min at RT (dark). Cells were analyzed within 1h by LSR II flow cytometer and FACS Diva software (Becton Dickinson). The results were computed as percent content of cells binding AnnV or/and 7-AAD among whole MSC fraction.

**Caspase-3 and -7 activity assay**

Cell apoptosis was detected by employing Vybrant FAM Caspase-3 and 7- Assay Kit (Life technologies) with flow cytometry. Thus, cells from all tested groups re-suspended in DMEM/F12 were incubated with 30X FLICA reagent for 60 min at 37^o^C and 5%CO_2_ accordingly to manufacturer’s protocol. Cells were subsequently washed twice with 1X Wash Buffer, re-suspended in DPBS and co-stained with Hoechst 33342 for 30min at 37^o^C. Cells were analyzed by employing LSR II flow cytometer and FACS Diva software (Becton Dickinson). The results were computed as percent content of cells exhibiting caspase-3 and -7 activity among whole MSC fraction.

**Global proteomic analysis -sample processing**

**Preparation of protein samples for proteome analysis**

Cells from all tested groups (1.7 x 10^6^) were lysed in 170 µl of 4% SDS (BioShop) and 0.1 M DTT (BioShop) in Tris-HCl (BioShop) pH 7.6 and sonicated for 10 min (320W, 30s on/off), using Bioruptor UCD-200 (Diagenode) for total protein harvesting. The sample were further incubated at 95°C for 5 min and centrifuged at 30,000xg for 30 min at 20°C.

**Filter Aided Sample Preparation for LC-MS/MS analysis**

The filter assisted sample preparation (FASP) method was performed based on procedures described by Wisniewski et al . Lysates were diluted by 8M urea (BioShop) in 50 ammonium bicarbonate (300 μl) (Fluka), reduced with DTT (final concentration was 50mM, 15 min) and applied on the 30 kDa cut-off filter (Vivacon 500, Sartorius Stedim). After centrifugation (14,000xg, two-times, 15 min, RT) the proteins were washed 200μl of 8M urea and centrifuged (14,000×g, two-times 45 min, RT). The proteins were alkylated with iodoacetamide (final concentration was 0.1 mg/ml in 8 M urea, 20 min, in the dark) (BioShop). The samples were centrifuged and subsequently washed three times with 8M urea (14,000×g, two-times 25 min, RT) and four times with 50 mM ammonium bicarbonate (14,000×g, two-times 20 min, RT). After the last centrifugation step, 75 μl of ammonium bicarbonate containing 2.5μg of trypsin (Promega) was added. After on-filter protein digestion (overnight, 37 °C) the resulting peptides were spin down (14,000 × g, 30 min, 25°C) and the filter unit was washed two times by 40 μl of ammonium bicarbonate and additionally with 50 μl 0.5M NaCl (14,000×g, 30 min, 25°C). Afterwards 2 μl of 100% trifluoroacetic acid (TFA; JT Baker) was added, peptide samples were centrifuged (35,000×g, 20min, 4°C) and moved into vial insert prior to LC-MS/MS analysis.

**Evaluation of protein concentration**

The peptide content was estimated by UV light spectral density at 280 nm (Nano-Drop, Thermo Scientific) using an extinctions coefﬁcient of 1.1 of 0.1% (g l^–1^) solution that was calculated on the basis of the frequency of tryptophan and tyrosine in vertebrate proteins .

**Liquid chromatography and tandem mass spectrometry (LC-MS/MS)**

Peptides were analyzed by mass spectrometry (MS) using an UltiMate 3000RS LC nanoSystem (Dionex) coupled with Q-Exactive mass spectrometer (Thermo Fisher Scientific) with DPV-550 Digital PicoView nanospray source. 1.5µg of peptides were injected on a C18 precolumn (Acclaim PepMap Nano trap Column) using 2% acetonitrile (JT Baker) with 0.05% TFA as a mobile phase, and further separated on a 50cm × 75μm RP column (Acclaim PepMap 75 μm 100 Å Nano Series TM Column) with gradient 2–40% ACN in 0.05% formic acid (FA; Sigma-Aldrich) for 360 minutes. The electrospray voltage was 2.2kV and the ion transfer tube temperature was 250°C. The Q-Exactive was operated in data dependent mode using “fast method” based on methods published by *Kelstrup et al*. with slightly modifications. Full MS scans were acquired in the Orbitrap mass analyzer over m/z 300–2000 range with resolution 70,000 (at m/z  200). The target value was 1.00E+06. The top twelve most intense peaks with charge state ≥ 2 were fragmented in the HCD collision cell normalized collision energy of 27% (the isolation window was 1.2 m/z). Tandem mass spectrum was acquired in the Orbitrap mass analyzer with resolution 17,500 at m/z 200. The target value was 5.00E+05. The ion selection threshold was 1.10E+05 counts, and the maximum ion accumulation times for the survey scan and the MS/MS scans were 120 ms and 60 ms respectively, dynamic exclusion was set to 30s.

**Analysis of proteomic data**

Peaklists from RAW files were generated by Proteome Discoverer platform 1.4 (Thermo Fisher Scientific) and searched against SwissProt database with Rodentia taxonomy restriction (release May 2014, 26 248 sequences) using locally installed MASCOT search engine (v.2.4.0, Matrix Science). The following search parameters were applied: up to one missed cleavages allowed for full tryptic digestion, precursor mass tolerance: 10 ppm, product ions mass tolerance: 0.02 Da, fixed modification: carbamidomethylation (C), variable modifications: oxidation (M), deamidated (NQ), phosphorylation (STY). For protein identification only high confidence peptides were taken, what means that Targed False Discovery Rate (FDR) less than 1% for peptide identification was assumed.

Quantitative analysis of common proteins was performed. Comparison was made in pairs: MSCs carrying MCPIP1 vs. Puro-treated MSCs and MSCs carrying MCPIP1 vs. untreated MSCs. As it was shown that distributing shared spectral counts (dNSAFs) based on the number of unique spectral counts led to the most accurate and reproducible results for protein label-free proteomic quantification in broad dynamic range we used it to compare the amount of the particular protein (k) in the subset of co-occurring proteins:

Distribution factor *d*:

where: dNSAF, spectral counts from shared peptides are distributed among protein isoforms based on a distribution factor, *d*. Spectral counts from peptides uniquely mapping to a protein are denoted as “uSpC”, while spectral counts from peptides shared between isoforms are labeled “sSpC”. Protein amino acid lengths mapping to unique and shared peptides are denoted as “uL” and “sL”, respectively.

Proteins were classified as differentially expressed in compared cells lines if dNAFs fold change of average value of dNSAF was higher than 2.0. The proteins which were identified at least in 1 of 2 samples of one group on the basis at least 2 unique peptides but did not occur in any sample of second group were considered as differentially expressed.

**Capillary-like tube formation assay**

Capillary-like tube formation assay was performed by following protocol published by Arnaoutva et al . Thus, 24-well plates were coated with Matrigel Matrix Grow Factor Reduced (BD Pharmingen) (100µl/well) and incubated at 37^o^C for 30 min. MCPIP1-expressing MSCs as well as Puro-treated and untreated cells were seeded at a density of 1x10^5^ cells/well in the EGM-2MV medium (Clonetics, Lonza). HUVECs were used as a positive control while freshly isolated total nucleated cells (TNCs) from murine BM were utilized as negative control. Cell were incubated for 12 or 18h in 37^o^C, 5% CO_2._. The tube formation process was investigated every 2h and images of all cells were collected with Olympus IMT-2 microscope equipped into CCD camera. The results were computed as absolute number of capillary-like structures and branches formed by cultured cell groups at different time points along the assay.

**Cardiac and angiogenic differentiation**

To examine differentiation potential of MCPIP1-expressing MSCs when compared to Puro-treated and untreated MSCs, cells were cultured in specific differentiation- stimulating media at 72h post transduction. Thus, the MSCs were detached with 0.25% Trypsin/EDTA, washed with DPBS and seeded at density 0.8x10^4^ cells/ cm^2^ on cell culture dish (BD Falcon). Two distinct differentiation protocols were used as described below.

**Cardiomyogenic differentiation**

Cells were cultured at dish coated with 50 µg/ml collagen type I (Sigma-Aldrich) in DMEM/F12 with 4mM L-glutamine, 4.5g/L glucose, 2% FBS and 10ng/ml bFGF, 10ng/ml VEGF and 10 ng/ml TGFβ1 (all grow factors from R&D Systems). The grow factors were supplemented daily and whole medium was replaced every 2 days. Cells were examined for cardiac differentiation at 5 and 10 days of culture. The expression of selected cardiac- specific markers was evaluated on both mRNA and protein levels.

**Endothelial differentiation**

Cells were cultured at dish coated with both fibronectin (50 µg/ml, Corning) and gelatin (0.1%, Sigma-Aldrich) in EGM-2MV endothelial medium (Lonza). EGM-2MV was replaced every 2 days. Cells were examined for angiogenic differentiation at 5 and 10 days of culture. The expression of selected endothelial- specific markers was evaluated on both mRNA and protein levels. Functional capillary-like forming assay was also performed with MSCs following 5 and 10 days of angiogenic differentiation.

Expression of selected autophagy- related genes was also evaluated in MSCs following 5 and 10 days of cardiac or angiogenic differentiation culture.

**Gene expression analysis by real-time RT-PCR**

Total RNA was isolated from MCPIP1-expressing MSCs, Puro-treated and untreated MSCs by employing GeneMATRIX Universal RNA Purification Kit (EURx). RNAs were treated with RNase-free DNase I (1U; Life Technologies) to remove DNA contamination. 200ng of total RNA was used to reverse transcription by TaqMan Reverse Transcription Reagents (Life Technologies) and reactions were performed according to manufacturer’s protocol: 1 cycle at 25^o^C for 10 min, 1 cycle at 48^o^C for 30 min and finally 1 cycle at 95^o^C for 5 min.

Expression of selected murine genes related to pluripotency state (Oct-3/4A, Sox2, Klf4, c-Myc), cardiac (Gata-4, Nkx2.5, Myl2, Myh6) and endothelial (Gata-2, Tie-2, VE-cadherin, vWF) differentiation as well as autophagy (Beclin 2, Atg7) were examined by real-time PCR using an ABI PRISM 7000 sequence detection system (Applied Biosystems). β2-microglobulin was used as a control housekeeping gene.

Real-time PCR was performed using Sybr Green qPCR Master Mix (EURx), cDNA template (10ng), forward primer (1µM) and reverse primer (1µM; both from Genomed). The sequences of primers were included in table below. Reactions were performed under the following conditions: 1 cycle at 50^o^C for 2 min, 1 cycle at 95^o^C for 10 min, followed by 40 cycles at 94^o^C for 15 s, 60^o^C for 30 s, 72^o^C for 30 s. Relative quantification of genes expression was calculated using the comparative C_t_ method. The relative quantitative value of the target- normalized to an endogenous control (β2-microglobulin gene) and relative to a calibrator - was expressed as 2^-ΔΔCt^ (i.e. fold difference), where ΔCt = [Ct of target genes] – [Ct of endogenous control gene] and ΔΔCt = [ΔCt of samples for target genes] – [ΔCt of calibrator for target gene].

| **Sequences of primers employed in real-time PCR** | | |
| --- | --- | --- |
| Gen | Description | Sequences |
| B2-microglobulin | Housekeeping gene | (F) CATACGCCTGCAGAGTTAAGCA  (R) GATCACATGTCTCGATCCCAGTAG |
| MCPIP1 | Zinc finger CCCH-type containing 12A | (F) CAGCCTCGACCAGATGTGCC  (R) CAGCCGCTCCTCGATGAAGC |
| Klf4 | Kruppel-like factor 4 | (F) GACTAACCGTTGGCGTGAGG  (R) TAGGAGGGCCGGGTTGTTAC |
| c-Myc | Myelocytomatosis oncogene | (F) GTTGGAAACCCCGCAGACAG  (R) TCGTCGCAGATGAAATAGGGC |
| Oct4  (Pou5f1) | Pou5f1 POU domain, class 5, transcription factor 1 | (F) GCCTTTCCCTCTGTTCCGT  (R) TGTCTACCTCCCTTGCCTTGG |
| Sox-2 | SRY-box containing gene 2 | (F) GCTCGCAGACCTACATGAAC  (R) GCCTCGGACTTGACCACAG |
| Gata-4 | Cardiac transcription factor | (F) TCCAGTGCTGTCTGCTCTAAGC  (R) TGGCCTGCGATGTCTGAGT |
| Nkx2.5 | Cardiac transcription factor | (F) TTCAAGCCCGAGGCCTACTC  (R) AGCGCGCACAGCTCTTTTT |
| Myl2 | Myosin Light Chain 2 | (F) GAACAGAGACGGCTTCATCGA  (R) TCGTCCTAGGGCAGCAAATG |
| Myh6 | Myosin Heavy Chain 6 | (F) ACTGTGGTGCCTCGTTCCA  (R) TGTCACTCAAACTCTGGTTAACTTTTC |
| Gata-2 | Endothelial Transcription Factor | (F) TGCACAATGTTAACAGGCCAC  (R) CCTCGAAACATTCAGCCCCT |
| Tie-2 | Angiopoietin-1 Receptor | (F) AGCGTCTATCGGACTCCCTC  (R) TGCTCCCTGGGGACTAAGTT |
| VE- cadherin | Endothelial-Specific Cadherin | (F) CACGGACAAGATCAGCTCCT  (R) ACATAGTGGGGCAGCGATTC |
| vWF | Von Willebrand factor | (F) GGCAAAACAAGCCTGCATGA  (R) ATAGCGCCGTAGATGCTGAC |
| Beclin 2 | Autophagy- related gene | (F) CATGGAGGGGTCTAAGGCG  (R) TTAGCCTCTTCCTCCTGGGTC |
| Atg7 | Autophagy- related gene | (F) AATGCATACAGGCCTCTGGAAAA  (R) GAAAGCCTCATGGCAGGAAA |

**Immunocytochemistry**

To evaluate i) Gata-4 and troponin T-C expression in cells differentiated into cardiomyocytes as well as ii) Gata-2 and VE-cadherin expression in endothelial differentiation, immunocytochemistry staining were performed. Thus, at 5 and 10 day of differentiation culture, medium was removed and cells were washed with DPBS and fixed with 4% paraformaldehyde (Sigma-Aldrich) for 20 min (RT). Cells were subsequently permeabilized with 0.1% Triton X-100 solution (Sigma-Aldrich) for 8 min (RT) and washed with DPBS. Cells were stained against: i) cardiac- specific proteins – with primary anti-Gata-4 antibody (mouse monoclonal IgG_2a_, 1:50) and anti-troponin T-C antibody (goat polyclonal IgG, 1:50); ii) endothelial- specific proteins – with primary anti-Gata-2 (rabbit polyclonal IgG, 1:50) and anti-VE-cadherin (mouse monoclonal IgG_1_, 1:20) for 16h at 4^o^C. Following secondary antibodies were subsequently added for cardiac (i) and endothelial (ii) markers detection, respectively: i) donkey anti-mouse IgG antibody conjugated with Alexa Fluor 488 (1:250) and donkey anti-goat conjugated with Alexa Fluor 546 (1:250) and ii) goat anti-mouse IgG antibody conjugated with Alexa Fluor 546 (1:250) and goat anti-rabbit IgG antibody conjugated with Alexa Fluor 488 (1:250). The staining with secondary antibodies was performed for 2 h in 37^o^C. Cells were further washed with DPBS and nuclei were stained with 4’,6-Diamidino-2-phenylindole (DAPI, 2µM, Life Technologies,) for 15min in 37^o^C. VECTASHIELD Mounting Medium (Vector Laboratories) was used to mount coverslip. The preparations were analyzed with Leica DM IRE2 (Ver. 4000) fluorescent microscope under total 20x magnification (Leica Microsystems GmbH).

Detailed description of used antibodies is included in table below.

| **List of primary and secondary antibodies applied for immunocytochemistry staining** | | |
| --- | --- | --- |
|  | **Primary Antibodies** | **Secondary Antibodies** |
| Cardiomyogenic differentiation | Anti-Gata-4 (mouse monoclonal IgG_2a_) Santa Cruz Biotechnology, sc-25310 | Donkey anti-mouse Alexa Fluor 488 Jackson ImmunoResearch, 800-367-5296 |
|  | Anti-Troponic T-C (goat polyclonal IgG) Santa Cruz Biotechnology, sc-8121 | Donkey anti-goat Alexa Fluor 546 Life technologies, A11056 |
| Endothelial differentiation | Anti-Gata-2 (rabbit polyclonal IgG) Santa Cruz Biotechnology, sc-9008 | Goat anti-rabbit IgG Alexa Fluor 488 Life technologies, A11008 |
|  | Anti-VE-cadherin (mouse monoclonal IgG_1_) Santa Cruz Biotechnology, sc-9989 | Goat anti-mouse IgG Alexa Fluor 546 Life technologies, A11003 |

**Secretome analysis**

**Western blotting (semi-quantitative)**

MSCs on passage 3 or 4 were seeded on 24-well plate coated with 0.1% gelatin at concentration of 24x10^3^cell/well and transduced as previously described. At 72h following second transduction, MCPIP1-expressing MSCs as well as reated and untreated MSCs (Control) were cultured in EGM-2MV medium for 10 days to stimulate endothelial differentiation and subsequently were washed twice with DMEM/F12 and further cultured in DMEM/F12 with 0.5%BSA (Sigma-Aldrich) for 24h. Cell culture supernatants were collected and frozen at -80^0^C up to analysis. Semi-quantitative analysis of expression profile of 53 angiogenesis-related proteins was performed by employing Proteome Profiler Mouse Angiogenesis Array Kit (R&D Systems) according to manufacturer’s protocol. The nitrocellulose membranes were exposed to X-ray film for 1-2 min by MicroChemi (DNR Bio- Imaging System Ltd.) to visualize the assay. The average signals (pixel density) computed from duplicate spots representing each angiogenesis-related protein were determined by Quantity One software application (BioRad). An average background signal was subtracted from each spot during the analysis.

**Multiplex analysis (quantitative)**

Moreover, the cell culture supernatants were evaluated for presence of selected angiogenesis- related factors with Luminex- based platform. Quantitative analysis of concentration of endoglin, endothelin, VEGF-α, HGF, SDF-1, MCP-1 and IL-1β was performed using Milliplex MAP Kit (Mouse Angiogenesis/Growth Factor Magnetic Bead Panel; EMD Millipore) according to manufacturer’s protocol. The proteins concentrations were read on Luminex platform (Austin). Median Fluorescent Intensity (MFI) for each protein was analyzed using 5-parameter logistic or spline curve-fitting method for calculating concentrations in tested samples by employing Luminex IS 2.3 Software (Austin).

**Statistical analysis**

All experiments were performed at least in triplicates (except global proteome analysis, which was repeated twice). In every repetition of experiment was used primary culture of MSCs at passage 3-4. Results are presented as mean value ± standard deviation (SD). Comparison between MCPIP1-overexpressing MSCs and Puro-treated were performed by paired Student *t* tests. Untreated control cells (Control) were used in all experiments as a negative control. The results with P-values <0.05 were considered as statistically significant. All statistical analyses were performed using the Origin (ver. 9.1) statistical software (Microcal Software).

**References for Extended Materials and Methods**

1. Lipert B, Wegrzyn P, Sell H, Eckel J, Winiarski M, Budzynski A, et al. Monocyte chemoattractant protein-induced protein 1 impairs adipogenesis in 3T3-L1 cells. Biochim Biophys Acta. 2014;1843: 780-788.

2. Kitamura T, Koshino Y, Shibata F, Oki T, Nakajima H, Nosaka T, et al. Retrovirus-mediated gene transfer and expression cloning: powerful tools in functional genomics. Exp Hematol. 2003;31: 1007-1014.

3. Mahmood T, Yang PC. Western blot: technique, theory, and trouble shooting. N Am J Med Sci. 2012;4: 429-434.

4. Mizgalska D, Wegrzyn P, Murzyn K, Kasza A, Koj A, Jura J, et al. Interleukin-1-inducible MCPIP protein has structural and functional properties of RNase and participates in degradation of IL-1beta mRNA. FEBS J. 2009;276: 7386-7399.

5. Wisniewski JR, Zougman A, Nagaraj N, Mann M. Universal sample preparation method for proteome analysis. Nat Methods. 2009;6: 359-362.

6. Zhuang Y, Ma F, Li-Ling J, Xu X, Li Y. Comparative analysis of amino acid usage and protein length distribution between alternatively and non-alternatively spliced genes across six eukaryotic genomes. Mol Biol Evol. 2003;20: 1978-1985.

7. Kelstrup CD, Young C, Lavallee R, Nielsen ML, Olsen JV. Optimized fast and sensitive acquisition methods for shotgun proteomics on a quadrupole orbitrap mass spectrometer. J Proteome Res. 2012;11: 3487-3497.

8. Zhang Y, Wen Z, Washburn MP, Florens L. Refinements to label free proteome quantitation: how to deal with peptides shared by multiple proteins. Anal Chem. 2010;82: 2272-2281.

9. Arnaoutova I, George J, Kleinman HK, Benton G. The endothelial cell tube formation assay on basement membrane turns 20: state of the science and the art. Angiogenesis. 2009;12: 267-274.

10. Kucia M, Reca R, Campbell FR, Zuba-Surma E, Majka M, Ratajczak J, et al. A population of very small embryonic-like (VSEL) CXCR4(+)SSEA-1(+)Oct-4+ stem cells identified in adult bone marrow. Leukemia. 2006;20: 857-869.
