# Supplementary figures and images for: Monocyte Chemoattractant Protein-Induced Protein 1 (MCPIP1) Enhances Angiogenic and Cardiomyogenic Potential of Murine Bone Marrow-Derived Mesenchymal Stem Cells
Source: PLoS One. 2015 Jul 27;10(7):e0133746. doi: 10.1371/journal.pone.0133746 (PMC4516329; doi:10.1371/journal.pone.0133746)

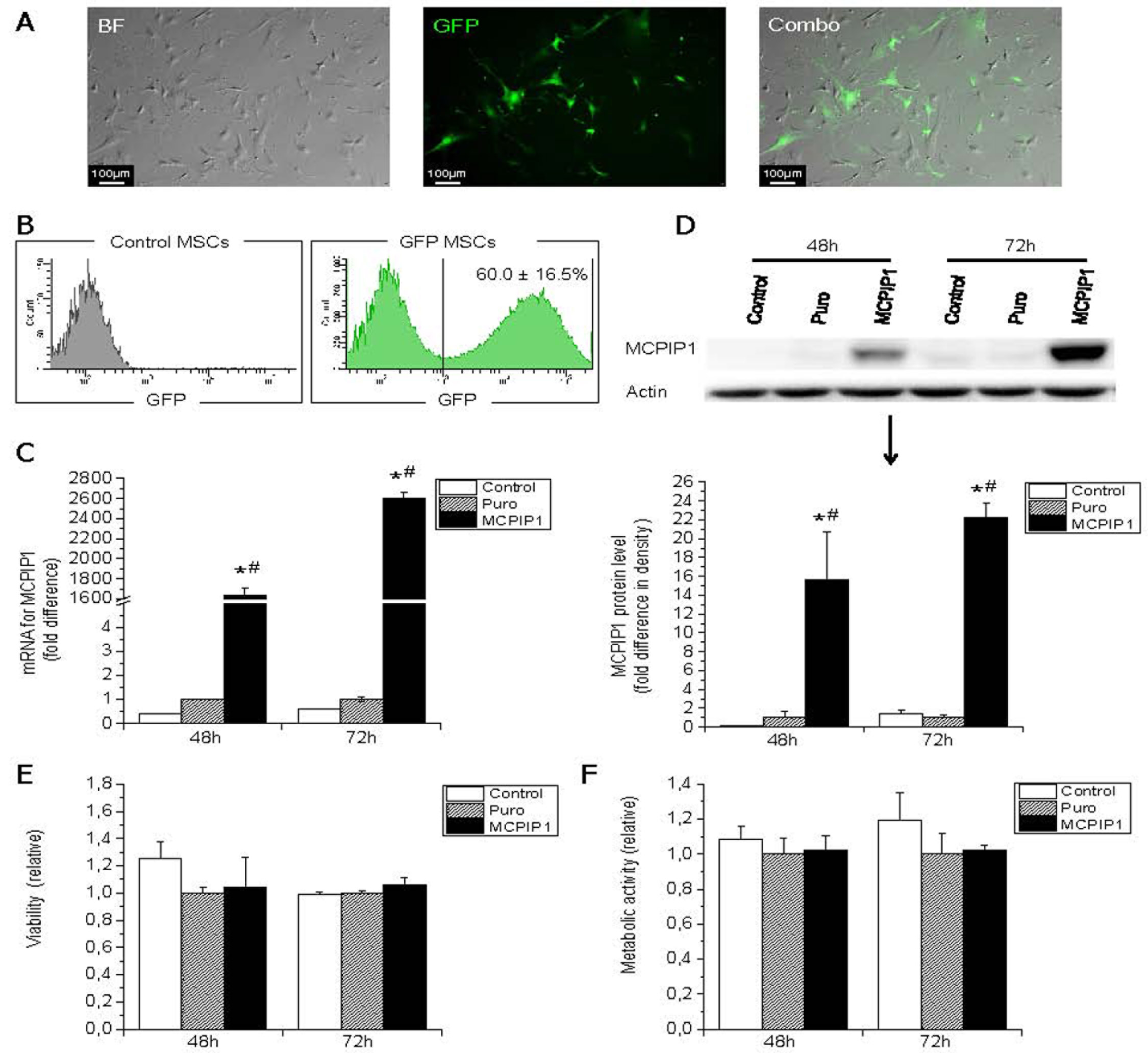

Supplement: S1 Fig — Morphology of the entire population of BM- derived MSCs following transduction with a vector carrying GFP by fluorescence (FL) microscopy. BF—Brightfield image, green FL—GFP- expressing MSCs, combo—combined image. Scale bars: 100 μm. (B) Representative histograms visualizing average values of transduction efficiency with pMX-based retroviral vector carrying GFP in the whole MSC population when compared with non-transduced Control MSCs. (C) Quantitative analysis of mRNA concentration for MCPIP1 at 48h and 72h following retroviral transduction. Black bars represent MSCs transduced with vector carrying MCPIP1 sequence, while hatched and white bars show MSCs transduced with empty vector (Puro) or untreated (Control) MSCs, respectively. The expression of MCPIP1 in Puro-treated cells is set as 1. (D) MCPIP1 overexpression in murine BM- derived MSCs. Upper panel: Representative Western blotting analysis of MCPIP1 protein expression at 48 and 72h following transduction. Actin was used as endogenous control. Lower panel: Semiquantitative assessment of MCPIP1 protein and actin concentrations based on pixel density analysis with Quantity One software. (E) Viability of MCPIP1-overexpressing MSCs when compared with Puro-treated cells (set as 1) at 48 and 72h following transduction by MTT assay. (F) Metabolic activity of MCPIP1-overexpressing MSCs when compared with Puro-treated cells (set as 1) assessed by ATP concentration measurement. All results are presented as mean ± SD. Statistically significant differences (P<0.05) are shown in comparison with Puro (*) and untreated Control (#) cells. Analysis based on three independent experiments. Control—untreated MSCs; Puro—empty vector-treated MSCs; MCPIP1- MSCs overexpressing MCPIP1. (TIF) [file pone.0133746.s002.tif]

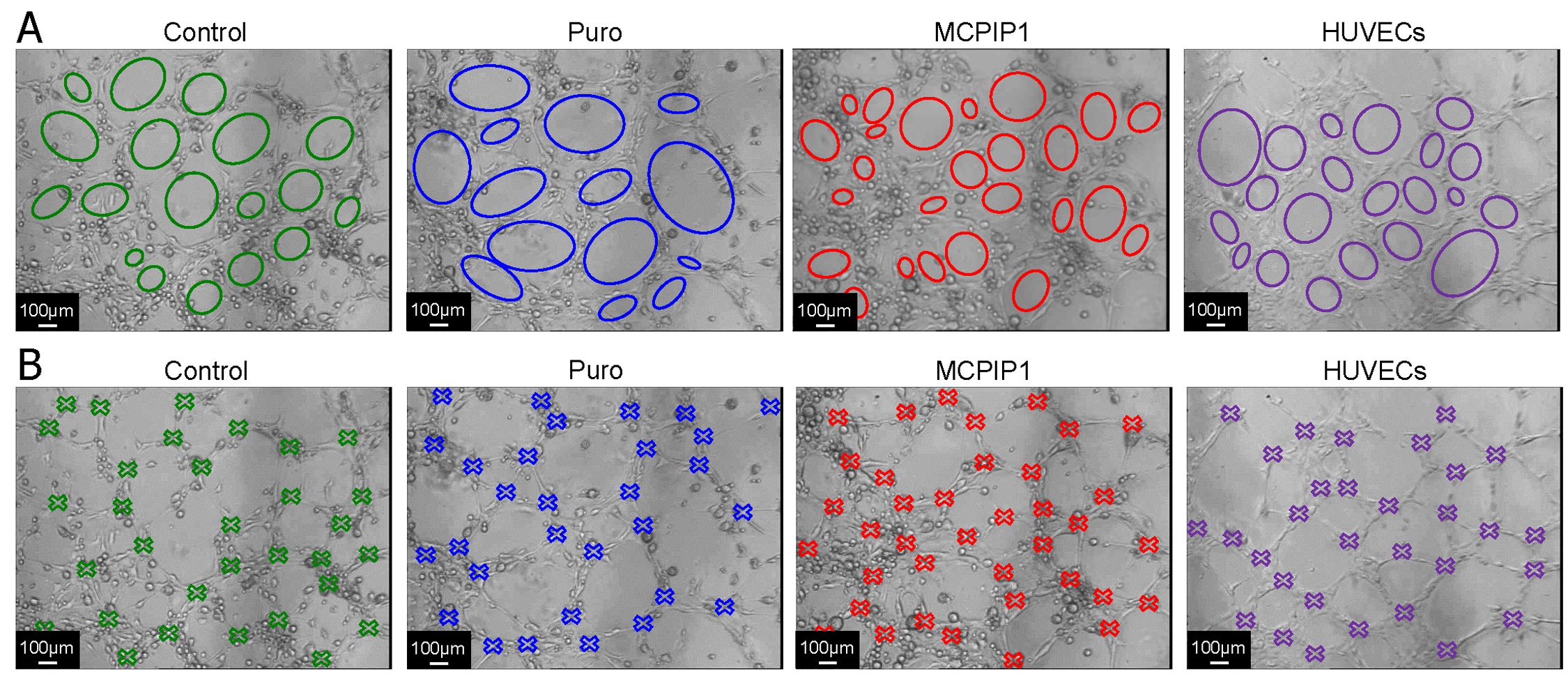

Supplement: S2 Fig — Six representative brightfield images of high-power fields (objective magnification 4x) were randomly selected and taken at every experimental timepoint for quantitative assessment. (A) Total number of capillaries were counted as shown by circles. (B) Total number of branches were assessed as shown by crosses. Average mean and SD were computed for every experimental timepoint. Control—untreated MSCs; Puro—empty vector-treated MSCs; MCPIP1- MSCs overexpressing MCPIP1. (TIF) [file pone.0133746.s003.tif]

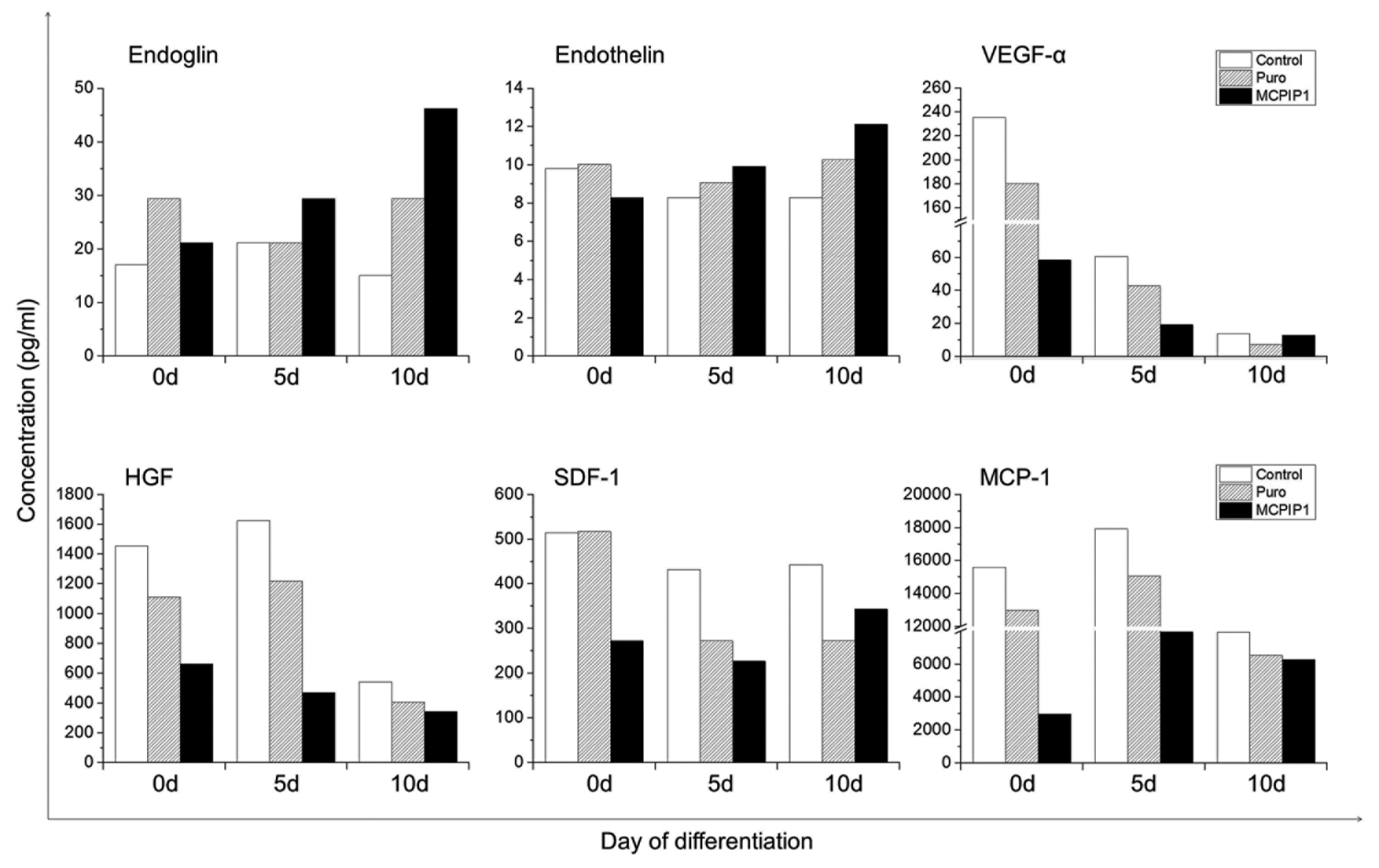

Supplement: S3 Fig — Concentration of analytes was evaluated in cell culture supernatants harvested from cultures of all three experimental groups of MSC (MCPIP1-overexpressing MSCs, empty vector- treated (Puro) MSCs and untreated (Control) MSCs) with Luminex xMAP technology using Mouse Angiogenesis/ Growth Factor Magnetic Bead Panel. Control—untreated MSCs; Puro—empty vector-treated MSCs; MCPIP1- MSCs overexpressing MCPIP1. (TIF) [file pone.0133746.s004.tif]
