# Supplementary material for: Monocyte Chemoattractant Protein-Induced Protein 1 (MCPIP1) Enhances Angiogenic and Cardiomyogenic Potential of Murine Bone Marrow-Derived Mesenchymal Stem Cells
Source: PLoS One. 2015 Jul 27;10(7):e0133746. doi: 10.1371/journal.pone.0133746 (PMC4516329; doi:10.1371/journal.pone.0133746)
Supplement: S1 Table — (DOC) [file pone.0133746.s005.doc]

**S1 Table.** **Quantitative analysis of number of branches and capillaries formed by MSCs in capillary-like formation assay - number of branches and capillaries calculated per field formed by non-differentiated MSC groups (at 72h following transduction).** All results are presented as mean (± SD) numbers per microscopic field**.** Analysis was performed three times with samples prepared from three independent experiments. Control - untreated MSCs; Puro - empty vector-treated MSCs; MCPIP1- MSCs overexpressing MCPIP1.

| **Absolute numbers of branches and capillaries at 72h following transduction/ field** | | | | | | | | | | | | |
| --- | --- | --- | --- | --- | --- | --- | --- | --- | --- | --- | --- | --- |
| No. of branches  (Mean ± SD) | | | | | |  | No. of capillaries  (Mean ± SD) | | | | | |
|  | **Control** | **Puro** | **MCPIP1** | **HUVEC** | **BM** |  |  | **Control** | **Puro** | **MCPIP1** | **HUVEC** | **BM** |
| **2h** | 1.9 ± 3.3 | 1.5 ± 2.5 | 1.9 ± 3.2 | 15.9 ± 7.1 | 0.0 ± 0.0 |  | **2h** | 0.1 ± 0.3 | 0.0 ± 0.0 | 0.0 ± 0.0 | 2.1 ± 3.1 | 0.0 ± 0.0 |
| **4h** | 19.8 ± 7.6 | 15.8 ± 3.1 | 27.1 ± 9.6 | 31.3 ± 9.0 | 0.0 ± 0.0 |  | **4h** | 4.3 ± 4.0 | 2.5 ± 2.3 | 7.7 ± 5.5 | 12.7 ± 9.0 | 0.0 ± 0.0 |
| **6h** | 29.4 ± 6.2 | 31.8 ± 3.6 | 36.0 ± 3.6 | 46.3 ± 9.6 | 0.0 ± 0.0 |  | **6h** | 9.1 ± 3.3 | 9.3 ± 3.6 | 11.6 ± 4.0 | 17.3 ± 9.6 | 0.0 ± 0.0 |
| **8h** | 26.7 ± 9.5 | 31.6 ± 3.8 | 33.8 ± 9.2 | 48.7 ± 3.3 | 0.0 ± 0.0 |  | **8h** | 9.2 ± 4.4 | 11.0 ± 2.4 | 12.9 ± 4.8 | 18.0 ± 5.2 | 0.0 ± 0.0 |
| **10h** | 26.8 ± 4.7 | 30.0 ± 4.3 | 30.8 ±5.2 | 49.2 ± 3.0 | 0.0 ± 0.0 |  | **10h** | 7.6 ± 2.9 | 11.6 ± 2.9 | 11.8 ±2.4 | 19.4 ± 1.9 | 0.0 ± 0.0 |
