# Supplementary material for: Monocyte Chemoattractant Protein-Induced Protein 1 (MCPIP1) Enhances Angiogenic and Cardiomyogenic Potential of Murine Bone Marrow-Derived Mesenchymal Stem Cells
Source: PLoS One. 2015 Jul 27;10(7):e0133746. doi: 10.1371/journal.pone.0133746 (PMC4516329; doi:10.1371/journal.pone.0133746)
Supplement: S2 Table — (DOC) [file pone.0133746.s006.doc]

**S2 Table.** **Quantitative analysis of number of branches and capillaries formed by MSCs in capillary-like formation assay - number of branches and capillaries formed by MSC groups after 5 and 10 days of endothelial stimulation**. All results are presented as mean (± SD) numbers per microscopic field**.** Analysis was performed three times with samples prepared from three independent experiments. Control - untreated MSCs; Puro - empty vector-treated MSCs; MCPIP1- MSCs overexpressing MCPIP1.

| **Absolute numbers of branches and capillaries after 5 days of endothelial stimulation/ field** | | | | | | | | | | | | |
| --- | --- | --- | --- | --- | --- | --- | --- | --- | --- | --- | --- | --- |
| No. of branches  (Mean ± SD) | | | | | |  | No. of capillaries  (Mean ± SD) | | | | | |
|  | **Control** | **Puro** | **MCPIP1** | **HUVEC** | **BM** |  |  | **Control** | **Puro** | **MCPIP1** | **HUVEC** | **BM** |
| **2h** | 0.0 ± 0.0 | 0.0 ± 0.0 | 0.0 ± 0.0 | 14.2 ± 2.6 | 0.0 ± 0.0 |  | **2h** | 0.0 ± 0.0 | 0.0 ± 0.0 | 0.0 ± 0.0 | 0.0 ± 0.0 | 0,0 ± 0,0 |
| **4h** | 19.8 ± 3.9 | 23.4 ± 4.9 | 22.5 ± 4.6 | 37.2 ± 3.4 | 0.0 ± 0.0 |  | **4h** | 10.0 ± 3.0 | 11.4 ± 3.3 | 9.7 ± 3.3 | 18.8 ± 2.5 | 0.0 ± 0.0 |
| **6h** | 31.8 ± 4.8 | 29.0 ± 3.6 | 28.8 ± 3.6 | 45.0 ± 5.6 | 0.0 ± 0.0 |  | **6h** | 15.8 ± 2.5 | 12.6 ± 1.5 | 12.8 ± 1.5 | 25.0 ± 3.4 | 0.0 ± 0.0 |
| **8h** | 35.2 ± 4.4 | 31.0 ± 3.1 | 32.2 ± 1.9 | 49.0 ± 4.6 | 0.0 ± 0.0 |  | **8h** | 14.8 ± 3.4 | 14.4 ± 0.9 | 15.2 ± 2.3 | 29.8 ± 3.1 | 0.0 ± 0.0 |
| **10h** | 27.8 ± 4.8 | 30.6 ± 3.3 | 36.7 ± 5.3 | 46.6 ± 1.5 | 0.0 ± 0.0 |  | **10h** | 12.2 ± 2.6 | 15.2 ± 2.2 | 17.3 ± 4.1 | 23.8 ± 2.2 | 0.0 ± 0.0 |
| **12h** | 25.8 ± 4.3 | 27.2 ± 4.3 | 27.5 ± 4.2 | 38.4 ± 6.9 | 0.0 ± 0.0 |  | **12h** | 11.0 ± 3.2 | 11.8 ± 1.8 | 15.4 ± 1.9 | 21.0 ± 5.0 | 0.0 ± 0.0 |
| **14h** | 25.4 ± 1.3 | 25.0 ± 4.5 | 28.5 ± 4.8 | 37.4 ± 4.6 | 0.0 ± 0.0 |  | **14h** | 10.8 ± 1.3 | 11.6 ± 2.1 | 14.8 ± 3.5 | 20.4 ± 2.4 | 0.0 ± 0.0 |
| **16h** | 21.4 ± 1.7 | 23.2 ± 3.3 | 20.8 ± 2.8 | 32.0 ± 6.0 | 0.0 ± 0.0 |  | **16h** | 10.0 ± 1.6 | 9.2 ± 0.8 | 11.0 ± 2.0 | 17.6 ± 4.2 | 0.0 ± 0.0 |
| **18h** | 20.8 ± 2.6 | 17.4 ± 3.3 | 22.7 ± 7.1 | 31.2 ± 4.5 | 0.0 ± 0.0 |  | **18h** | 7.4 ± 1.1 | 7.2 ± 1.6 | 10.3 ± 4.4 | 17.2 ± 4.0 | 0.0 ± 0.0 |
| **Absolute numbers of branches and capillaries after 10 days of endothelial stimulation/ field** | | | | | | | | | | | | |
| No. of branches  (Mean ± SD) | | | | | |  | No. of capillaries  (Mean ± SD) | | | | | |
|  | **Control** | **Puro** | **MCPIP1** | **Huvec** | **BM** |  |  | **Control** | **Puro** | **MCPIP1** | **Huvec** | **BM** |
| **2h** | 0.0 ± 0.0 | 0.0 ± 0.0 | 0.0 ± 0.0 | 14.2 ± 2.6 | 0.0 ± 0.0 |  | **2h** | 0.0 ± 0.0 | 0.0 ± 0.0 | 0.0 ± 0.0 | 0.0 ± 0.0 | 0.0 ± 0.0 |
| **4h** | 13.2 ± 2.4 | 11.0 ± 1.2 | 4.8 ± 3.4 | 22.4 ± 5.0 | 0.0 ± 0.0 |  | **4h** | 2.8 ± 0.8 | 0.8 ± 1.3 | 1.0 ± 0.7 | 9.0 ± 2.6 | 0.0 ± 0.0 |
| **6h** | 17.8 ± 4.2 | 19.4 ± 4.5 | 24.0 ± 4.0 | 32.2 ± 2.4 | 0.0 ± 0.0 |  | **6h** | 6.8 ± 2.7 | 9.8 ± 2.2 | 10.0 ± 1.4 | 17.8 ± 3.7 | 0.0 ± 0.0 |
| **8h** | 36.4 ± 6.6 | 34.2 ± 9.5 | 45.8 ± 3.9 | 48.4 ± 2.7 | 0.0 ± 0.0 |  | **8h** | 20.0 ± 5.3 | 18.2 ± 7.8 | 29.2 ± 3.1 | 28.0 ± 5.4 | 0.0 ± 0.0 |
| **10h** | 35.6 ± 3.4 | 33.8 ± 6.5 | 40.6 ± 5.2 | 41.6 ± 3.1 | 0.0 ± 0.0 |  | **10h** | 23.2 ± 3.7 | 19.6 ± 5.4 | 28.2 ± 1.9 | 24.8 ± 1.5 | 0.0 ± 0.0 |
| **12h** | 33.0 ± 9.7 | 33.8 ± 8.9 | 39.3 ± 5.8 | 35.6 ± 2.0 | 0.0 ± 0.0 |  | **12h** | 19.6 ± 9.7 | 16.2 ± 6.1 | 21.7 ± 2.6 | 22.8 ± 2.8 | 0.0 ± 0.0 |
| **14h** | 32.0 ± 6.4 | 29.2 ± 4.6 | 36.3 ± 4.8 | 34.6 ± 4.3 | 0.0 ± 0.0 |  | **14h** | 19.4 ± 3.9 | 14.4 ± 4.4 | 20.0 ± 3.5 | 19.2 ± 3.1 | 0.0 ± 0.0 |
| **16h** | 28.6 ± 2.7 | 27.6 ± 5.6 | 31.3 ± 3.0 | 34.2 ± 1.8 | 0.0 ± 0.0 |  | **16h** | 17.8 ± 3.4 | 13.2 ± 4.5 | 15.8 ± 3.1 | 18.6 ± 2.6 | 0.0 ± 0.0 |
| **18h** | 23.6 ± 3.8 | 24.0 ± 2.1 | 25.7 ± 1.2 | 24.6 ± 3.4 | 0.0 ± 0.0 |  | **18h** | 14.0 ± 3.4 | 11.8 ± 3.3 | 15.8 ± 1.6 | 16.8 ± 4.1 | 0.0 ± 0.0 |
